# Aging‐Associated Liver Sinusoidal Endothelial Cells Dysfunction Aggravates the Progression of Metabolic Dysfunction‐Associated Steatotic Liver Disease

**DOI:** 10.1111/acel.14502

**Published:** 2025-02-06

**Authors:** Qingqing Dai, Quratul Ain, Navodita Seth, Hongchuan Zhao, Michael Rooney, Alexander Zipprich

**Affiliations:** ^1^ Department of Internal Medicine IV (Gastroenterology, Hepatology, and Infectious Diseases) Jena University Hospital Jena Germany; ^2^ Department of Hepatopancreatobiliary Surgery The First Affiliated Hospital of Anhui Medical University Hefei China; ^3^ Department of General Surgery The First Affiliated Hospital of Anhui Medical University Hefei China

**Keywords:** aging, fibrosis, liver sinusoidal endothelial cells, metabolic dysfunction–associated steatotic liver disease

## Abstract

Aging increases the susceptibility to metabolic dysfunction–associated steatotic liver disease (MASLD). Liver sinusoidal endothelial cells (LSECs) help in maintaining hepatic homeostasis, but the contribution of age‐associated LSECs dysfunction to MASLD is not clear. The aim of this study was to investigate the effect of aging‐associated LSECs dysfunction on MASLD. Free fatty acid–treated AML12 cells were co‐cultured with young and etoposide‐induced senescent TSEC cells to evaluate the senescence‐associated endothelial effects on the lipid accumulation in hepatocytes. In addition, young and aged rats were subjected to methionine‐choline‐deficient diet–induced metabolic dysfunction–associated steatohepatitis (MASH). Hepatic hemodynamics and endothelial dysfunction were evaluated by in situ liver perfusion. Liver tissue samples from young and aged healthy controls and MASH patients were also analyzed. Steatotic AML12 cells co‐cultured with young TSEC cells showed less lipid accumulation, and such effect was abolished by eNOS inhibitor or with senescent TSEC cells. However, co‐culture with resveratrol‐treated senescent TSEC cells could partially resume the NO‐mediated protective effects of endothelial cells. Furthermore, aged MASH rats showed more severe liver injury, steatosis, fibrosis, and endothelial and microcirculatory dysfunction. In addition, aged MASH patients showed more pronounced liver injury and fibrosis with lower hepatic eNOS, p‐eNOS, and SIRT1 protein levels than in young patients. Senescence compromises the protective effects of LSECs against hepatocyte steatosis. In addition, aging aggravates not only liver steatosis and fibrosis but also intensifies LSECs dysfunction in MASH rats. Accordingly aged MASH patients also showed endothelial dysfunction with more severe liver injury and fibrosis.

## Graphical abstract

Aged rats and human are more susceptible to MASLD‐related liver injury, steatosis, and fibrosis, accompanied by worsening of endothelial dysfunction. The SIRT1/p‐eNOS/NO pathway is involved in aggravated MASLD caused by aging‐related LSECs dysfunction. Therefore, decreased SIRT1 and LSECs dysfunction appear as attractive therapeutic targets to alleviate MASLD progression.

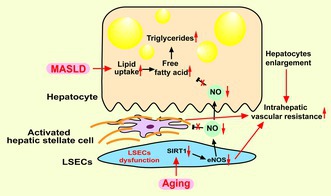

## 1 Introduction

Metabolic dysfunction–associated steatotic liver disease (MASLD) and advanced‐form metabolic dysfunction–associated steatohepatitis (MASH) have currently become the most common chronic liver diseases globally (Younossi et al. 2023). The improved healthcare leads to remarkable increment in human life expectancy, contributing to escalating morbidity of aging‐associated chronic and geriatric diseases (Iburg et al. 2023). Aging has been reported to accelerate the progression of MASLD and liver fibrosis (Li et al. 2022), but the underlying mechanisms are not yet fully understood. Liver sinusoidal endothelial cells (LSECs) receive increasing attention in liver diseases due to their distinctive location and function. During aging, LSECs undergo endothelial dysfunction and capillarization (Maeso‐Diaz et al. 2018), which impairs substance exchange and intercellular communications between LSECs with hepatocytes and hepatic stellate cells (HSCs).

Endothelial dysfunction is defined by the incapacity of blood vessels to dilate upon elevated blood flow, as evidenced by the reduced nitric oxide (NO) bioavailability due to endothelial NO synthase (eNOS) downregulation (Hammoutene and Rautou 2019). Cumulative studies have reported that LSECs dysfunction occurs in MASLD and promote liver steatosis and fibrosis. Dysfunctional LSECs disrupt intrahepatic microcirculation, contributing to increased intrahepatic vascular resistance and portal hypertension (Eccleston et al. 2011; Francque et al. 2012; Pasarin et al. 2012). Likewise, 4 weeks of methionine‐choline‐deficient (MCD) diet administration caused severe steatosis and significant elevation of intrahepatic vascular resistance and portal pressure in the absence of inflammation and fibrosis, while scanning electron microscopy of vascular casts showed compressed sinusoids and disorganized trabecular pattern (Francque et al. 2012). Pasarin et al. also observed an increased portal pressure in rats fed with 65% fat‐rich diet for 1 month, along with significantly reduced p‐eNOS protein expression and activity with low vasodilation response to acetylcholine validating the presence of LSECs dysfunction in MASLD (Pasarin et al. 2012). Besides, lipid accumulation in hepatocytes and HSCs activation are notable contributors to progression of MASLD/MASH (Hammoutene and Rautou 2019). Moreover, eNOS agonist and NO donor and precursor were previously found to significantly reduce hepatocytes lipid accumulation and HSCs activation in cell and animal experiments (Cordero‐Herrera et al. 2019; Fang et al. 2022; Garcia‐Villafranca, Guillen, and Castro 2003; Wang et al. 2013). Thus, the contribution of LSECs dysfunction to hepatocytes and HSCs deterioration in MASLD/MASH warrants more investigation.

Decreased levels of Sirtuin 1 (SIRT1), a crucial deacetylase that regulates multiple aging‐related processes is associated with the aggravation of alcohol‐ and carbon tetrachloride‐induced liver injury and fibrosis in aged liver (Adjei‐Mosi et al. 2023; Ramirez et al. 2017). LSECs are the earliest and predominant senescent cells in the liver, with the percentage of senescent LSECs increasing from < 1% at 2 months to > 50% at 2 years of age (Grosse et al. 2020). Recently, SIRT1 overexpression has been implicated in improving the LSECs defenestration in liver fibrogenesis by inhibiting the stress‐induced untimely LSECs senescence (Luo et al. 2021). However, the role of SIRT1 in aging‐related LSECs senescence and dysfunction has not been reported. Besides, previous studies have briefly explored the effects of aging on hepatic hemodynamics and the phenotype of the main hepatic cell types in healthy rats and rats with advanced chronic liver disease (Maeso‐Diaz et al. 2018, 2019). However, comprehensive reports of hepatic hemodynamics and microcirculation in young and aged normal and MASH rats are not available. In the present study, we thoroughly delineated the age‐ and MASH‐associated impacts on hepatic hemodynamics and microcirculation including portal vein, hepatic artery, and sinusoids in MCD diet–induced MASH rat model. In addition, we also explored the beneficial effects of SIRT1 on senescent LSECs and the effect of aging‐associated LSECs dysfunction on hepatocyte steatosis in in vitro experiments.

## 2 Results

### 2.1 Endothelial Dysfunction in Senescent TSEC Cells

**FIGURE 1:**
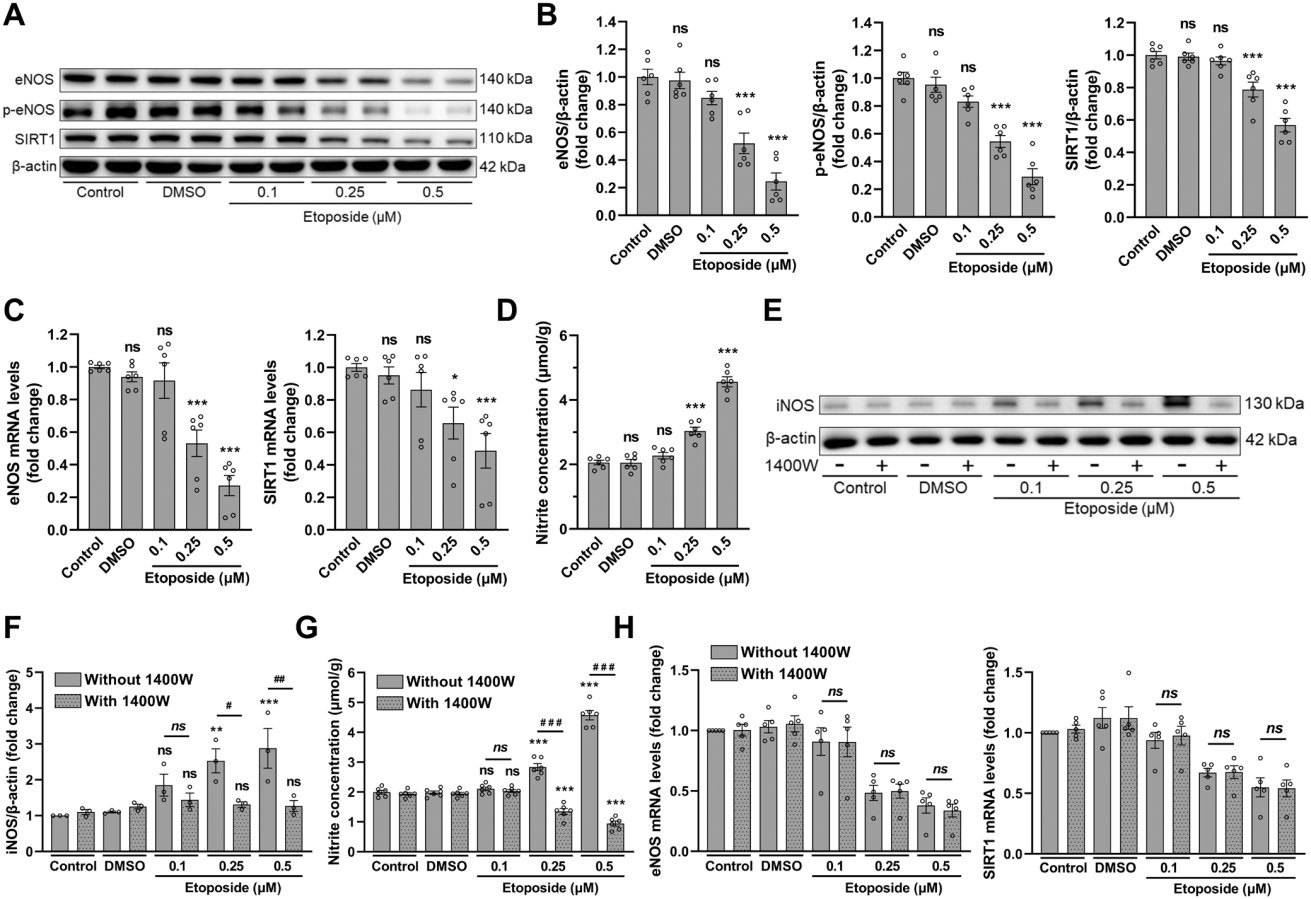
Endothelial dysfunction and decreased SIRT1 expression in senescent TSEC cells. (A) Western blot and (B) quantitative analysis of eNOS, p‐eNOS, and SIRT1 protein levels in senescent TSEC cells at day 5 (*N* = 6). (C) Quantitative analysis of senescent TSEC mRNA expression levels of eNOS and SIRT1 at day 5 (*N* = 6). (D) Nitrite concentration in control and etoposide‐treated TSEC cells at day 5 (*N* = 6). (E) Western blot and (F) quantitative analysis of iNOS protein levels after treatment with different concentration of etoposide in the presence or absence of iNOS inhibitor 1400 W (day 5) (*N* = 3). (G) Nitrite concentration in TSEC cells at day 5 after treatment with etoposide with/without 1400 W (*N* = 6). (H) Quantitative analysis of eNOS and SIRT1 mRNA levels in TSEC cells at day 5 after etoposide treatment in the absence or presence of 1400 W (*N* = 5). Data expressed as mean ± SEM, one‐way ANOVA followed by Tukey's post hoc test. In B–D, F–H, *, **, ***, ns represent comparison with control group; in F–H, #, ##, ###, *ns* show intergroup comparison. */#*p* < 0.05, **/##*p* < 0.01, ***/###*p* < 0.001, and ns/*ns* = not significant.

TSEC cells were treated with different concentrations of etoposide for 2 days (day 2) and then additionally cultured in fresh medium for another 3 days (day 5) to induce senescence. The cell viability was measured at day 2 and day 5 (Figure S1A,B). The results suggested excessive cytotoxicity in 0.5 and 1 μM etoposide‐treated cells, resulting in death of almost all cells at day 5. However, TSEC cells treated with 0.25 μM etoposide concentration showed growth arrest with cell viability of 62% at day 2 and 46% at day 5. Likewise, senescence‐associated β‐galactosidase (SABG) staining at day 5 also showed a concentration‐dependent increase in the percentage of SABG‐positive senescent cells (Figure S1C,D). Thus, 0.25 μM etoposide concentration was considered as the optimal concentration for senescence induction. Accordingly, the senescence marker p21 protein expression also increased dramatically at day 5 in 0.25 and 0.5 μM etoposide groups (Figure S1E,F). Moreover, the protein and/or mRNA expressions of eNOS, p‐eNOS, and SIRT1 decreased significantly at higher etoposide concentration (Figure 1A–C). These results demonstrated that endothelial function–related proteins and the antiaging SIRT1 were significantly decreased in senescent endothelial cells.

eNOS‐derived NO is important for endothelial function and vascular responses. Contrary to the decreased eNOS and p‐eNOS levels in 0.25 and 0.5 μM etoposide groups (Figure 1A–C), NO concentration significantly increased in both groups (Figure 1D). NO is originated from eNOS and inducible nitric oxide synthase (iNOS) in the liver (Iwakiri and Kim 2015), while etoposide treatment was previously reported to increase iNOS protein expression (Kwon et al. 2015). Therefore, we examined iNOS protein expression levels and found that it markedly increased in TSEC cells treated with higher etoposide concentration (Figure 1E,F). Accordingly, to exclude the involvement of etoposide‐induced iNOS in the detection of NO concentration, the highly selective iNOS inhibitor 1400 W (Garvey et al. 1997) was added during etoposide treatment. Correspondingly, etoposide‐induced iNOS protein levels and intracellular NO were greatly reduced by 1400 W treatment in 0.25 and 0.5 μM etoposide‐treated TSEC cells (Figure 1E–G), thus, indicating a remarkable decrease in eNOS‐derived NO concentration in senescent TSEC cells. Moreover, 1400 W supplementation did not influence the mRNA expression of eNOS and SIRT1 (Figure 1H).

### 2.2 Resveratrol Improved Senescence‐Associated Endothelial Dysfunction and Alleviated Cell Steatosis

**FIGURE 2:**
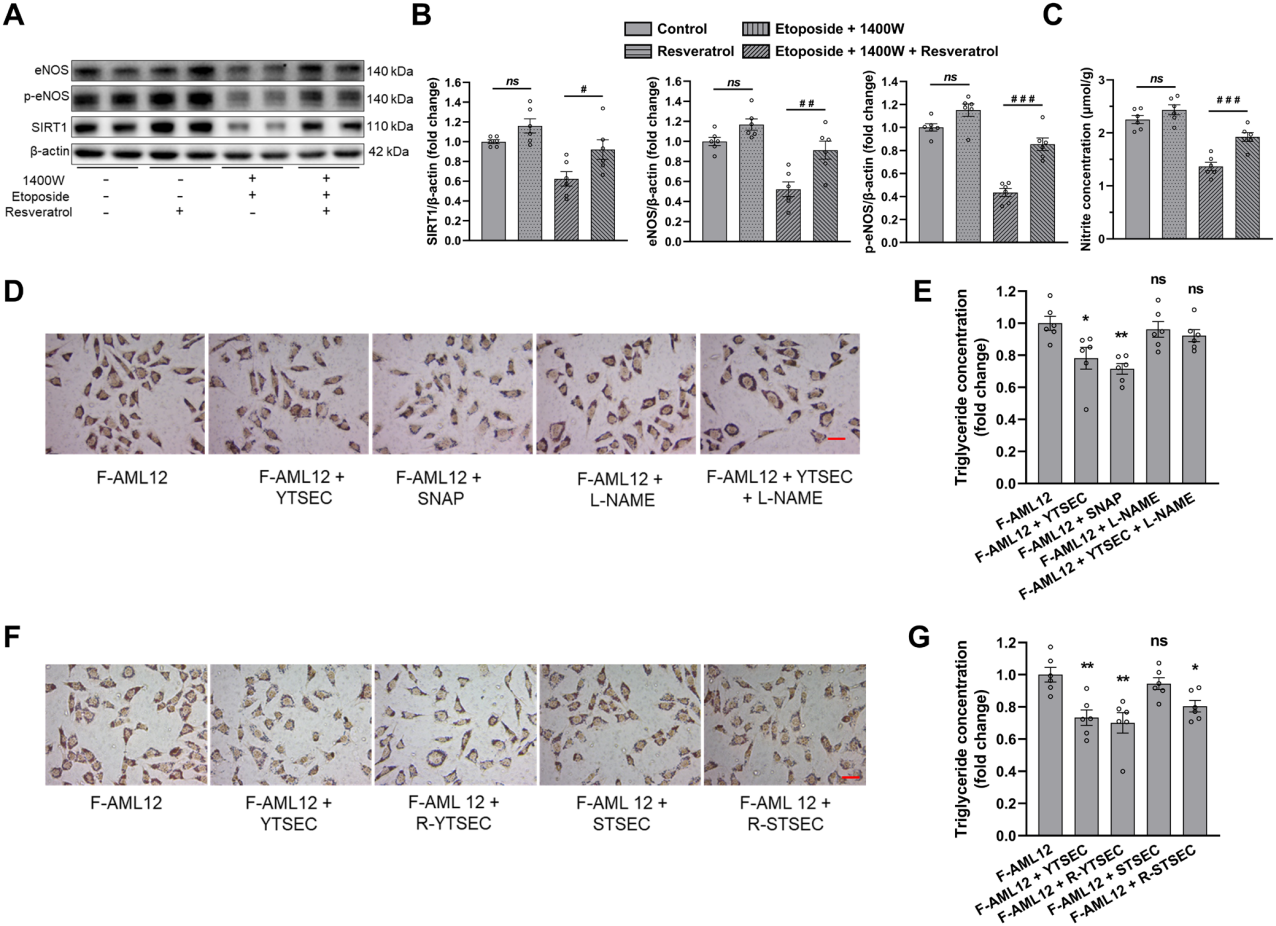
The effects of endothelial cells and resveratrol‐treated senescent endothelial cells co‐culture on hepatocyte fat accumulation. (A) Western blot and (B) quantitative analysis of eNOS, p‐eNOS, and SIRT1 protein levels of TSEC cells after etoposide and/or resveratrol treatment along with 1400 W. (C) TSEC nitrite levels after treatment with etoposide, resveratrol and 1400 W. (D) Oil Red O staining and (E) evaluation of triglyceride concentration in F‐AML12 mono‐culture or co‐cultured with YTSEC, mono‐culture with addition of nitric oxide donor SNAP, or inhibitor L‐NAME, or co‐cultured with Y‐TSEC in the presence of L‐NAME. (F) Oil Red O staining and (G) triglyceride concentration estimation in F‐AML12 cells cultured alone or co‐cultured with the indicated TSEC cells. In (D, F), scale bar = 5 μm. Data expressed as mean ± SEM (*N* = 6), one‐way ANOVA followed by Tukey's post hoc test. In (E) and (G), *, ** and ns show comparison vs. control group; in (B) and (C), #, ##, ###, *ns* show intergroup comparisons. */#*p* < 0.05, **/##*p* < 0.01, ***/###*p* < 0.001, and ns/*ns* = not significant. F‐AML12, high free fatty acid–treated AML12 cells; L‐NAME, NG‐Nitroarginine methyl ester hydrochloride; R‐STSEC, resveratrol‐treated senescent TSEC cells; R‐YTSEC, resveratrol‐treated young TSEC cells; SNAP, *S*‐Nitroso‐*N*‐acetyl‐DL‐penicillamine; STSEC, senescent TSEC cells; YTSEC, young TSEC cells.

Given the crucial role of SIRT1 in aging and cell senescence (Chen et al. 2020), we treated the etoposide‐induced senescent TSEC cells with SIRT1 activator resveratrol to observe whether SIRT1 activation could ameliorate senescence‐associated endothelial dysfunction. Firstly, we treated TSEC cells with different concentrations of resveratrol for 3 days to evaluate the resveratrol cytotoxicity. Resveratrol had no effect on TSEC viability at low concentrations up to 10 μM (Figure S2A). Therefore, 10 μM resveratrol was adopted for the treatment of senescent TSEC cells. Furthermore, resveratrol treatment had no additional effects on the etoposide‐induced iNOS (Figure S2B,C). Meanwhile, 1400 W was used in the senescent TSEC cells model to exclude the interference of etoposide‐induced iNOS on the eNOS‐derived NO (Figure S2D). Interestingly, resveratrol increased the protein levels of SIRT1, eNOS and p‐eNOS in senescent TSEC cells (Figure 2A,B). In addition, resveratrol elevated intracellular NO concentration in senescent TSEC cells where iNOS was simultaneously inhibited with 1400 W (Figure 2C).

AML12 cells were treated with low and high concentrations of free fatty acid (FFA) to induce steatosis. High FFA concentration treatment induced more pronounced lipid accumulation and higher triglyceride concentration (Figure S3A,B) and was therefore selected for subsequent experiments. Later, we co‐cultured young TSEC cells (without etoposide treatment) with FFA‐treated AML12 cells to explore the endothelial contribution on hepatocyte lipid accumulation. The results demonstrated reduced lipid accumulation and triglyceride concentration in co‐culture with young TSEC cells (Figure 2D,E). In addition, to examine the involvement of eNOS‐derived NO in hepatocyte steatosis, FFA‐treated AML12 cells were cultured in media supplemented with NO donor *S*‐Nitroso‐*N*‐acetyl‐DL‐penicillamine (SNAP) or co‐cultured with young TSEC cells in media containing eNOS inhibitor NG‐Nitroarginine methyl ester hydrochloride (L‐NAME). SNAP supplementation decreased lipid accumulation and triglyceride concentration, whereas addition of L‐NAME abolished young TSEC cells–mediated reduction in lipid accumulation and triglyceride concentration in FFA‐treated AML12 cells (Figure 2D,E). These results indicated that NO is an important regulator for lipid accumulation. Later, we found co‐culture with senescent TSEC cells diminished endothelial protection against hepatocyte fat accumulation, demonstrated by higher lipid buildup and triglyceride concentrations compared with young TSEC cells (Figure 2F,G). To further explore the previously established beneficial effects of resveratrol on endothelial function, we co‐cultured resveratrol‐treated TSEC cells with FFA‐treated AML12 cells. Likewise, resveratrol treatment in senescent TSEC cells partially restored the protection against AML12 cells fat accumulation (Figure 2F,G).

Besides, we used primary hepatocytes isolated from young rats and primary LSECs isolated from young and aged rats to validate the above results. High FFA concentrations induced more significant steatosis in primary hepatocytes (Figure S3C,D). No significant differences were observed in eNOS protein levels between LSECs isolated from young and aged rats before and after resveratrol treatment (Figure S4A,B). However, p‐eNOS and SIRT1 protein expression and NO levels were markedly decreased in aged primary rat LSECs. The protein expression levels of p‐eNOS and SIRT1 and NO levels increased noticeably after resveratrol treatment (Figure S4A–C). SNAP supplementation and co‐culture with young LSECs alleviated lipid deposition and triglyceride concentrations in high FFA concentrations‐induced steatotic primary hepatocytes, but the protective effect from young LSECs was abrogated by L‐NAME (Figure S4D,E). Similarly, the protective effect of LSECs against hepatocyte steatosis was substantially lost in steatotic primary rat hepatocytes co‐cultured with aged LSECs, but was partially reinstated by resveratrol treatment (Figure S4F,G).

### 2.3 Aging Aggravates MCD Diet–Induced Liver Injury and Endothelial Dysfunction

**FIGURE 3:**
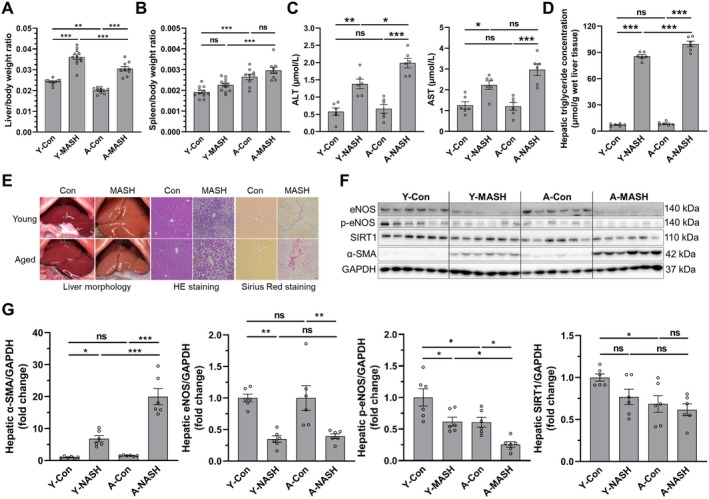
Age‐associated effects on liver injury and fibrosis of diet‐induced MASH model. (A) Liver/body weight ratio. (B) Spleen/body weight ratio. (C) Serum ALT and AST levels. (D) Hepatic triglyceride concentration. (E) Representative images of liver morphology, H & E staining, and Sirius Red staining (scale bar = 100 μm). (F) Western blot and (G) quantitative analysis of hepatic eNOS, p‐eNOS, SIRT1, and α‐SMA protein expression levels. Data expressed as mean ± SEM (*N* = 9–12 for A and B, *N* = 6 for C–G), one‐way ANOVA followed by Tukey's post hoc test, *p < 0.05, **p < 0.01, ***p < 0.001, and ns = not significant.

Metabolic dysfunction‐associated steatohepatitis model was developed by feeding young (8 weeks) and aged (78 weeks) rats with MCD diet for 12 weeks. The body weight curve, final body weight, Δ body weight (weight at start of the MCD diet − weight at end of the MCD diet), and liver and spleen weights are shown in Figure S5A–E. The liver/body weight ratios were significantly elevated in the MASH rats as compared to the control rats (Figure 3A). Likewise, the spleen/body weight ratios were higher in the MASH rats as compared to the age‐matched control rats albeit did not reach statistical significance; however, an age‐related increment was observed in spleen/body weight ratio for both control and MASH cohorts (Figure 3B).

Basal portal venous resistance (PVR) and sinusoidal vascular resistance (SVR) were markedly elevated in both young and aged MASH rats, and were at highest levels in aged MASH (A‐MASH) rats (Figure S6A,B). However, basal hepatic arterial resistance (HAR) was dramatically higher in young MASH (Y‐MASH) group than other three groups (Figure S6C), which could putatively be attributed to the low body weight of Y‐MASH rats. We subsequently performed experiments in weight‐matched young control rats with comparable body weight to Y‐MASH rats (Figure S6D), and no difference was observed in basal HAR between the two groups; however, the basal PVR and SVR were significantly higher in the Y‐MASH group (Figure S6E).

Biochemistry tests revealed that alanine aminotransferase (ALT) and aspartate aminotransferase (AST) levels were significantly higher in the MASH cohorts, while ALT levels were more pronounced in A‐MASH rats (Figure 3C, details in Table S1). Similarly, hepatic triglyceride concentrations were notably increased in the MASH rats but was more pronounced in A‐MASH rats (Figure 3D). Likewise, we observed a significant decrease in the gene expression of lipid metabolic genes such as sterol regulatory element binding protein 1c (SERBP1c), acetyl‐CoA carboxylase (ACC), and peroxisome proliferator‐activated receptor alpha (PPAR‐α) in A‐MASH rats as compared to the aged controls (A‐Con); however, an age‐related increase was observed in the expression of the lipid metabolic genes (Figure S7A). In addition, liver morphology and histology showed more severe liver injury, steatosis, and fibrosis in A‐MASH rats (Figure 3E), and hepatic α‐SMA protein expression was also significantly increased in A‐MASH rats (Figure 3F,G).

Moreover, eNOS protein levels were remarkably reduced in MASH rats compared with control rats, but age‐associated changes were not observed (Figure 3F,G). However, p‐eNOS protein levels were dramatically decreased in both MASH and aged rats, and was at lowest levels in the A‐MASH group (Figure 3F,G). In addition, age‐associated decline in SIRT1 protein levels was observed in control rats. However, SIRT1 protein level was reduced in MASH rats compared to the corresponding control rats but were not statistically different (Figure 3F,G). Interestingly, a remarkable disease‐related decline was observed in SIRT1 mRNA expression, while aging resulted in a nonsignificant increment of SIRT1 mRNA level in the control rats (Figure S7B).

### 2.4 LSEC Dysfunction, Liver Injury, and Fibrosis Exacerbate in Aged MASH Patients

**FIGURE 4:**
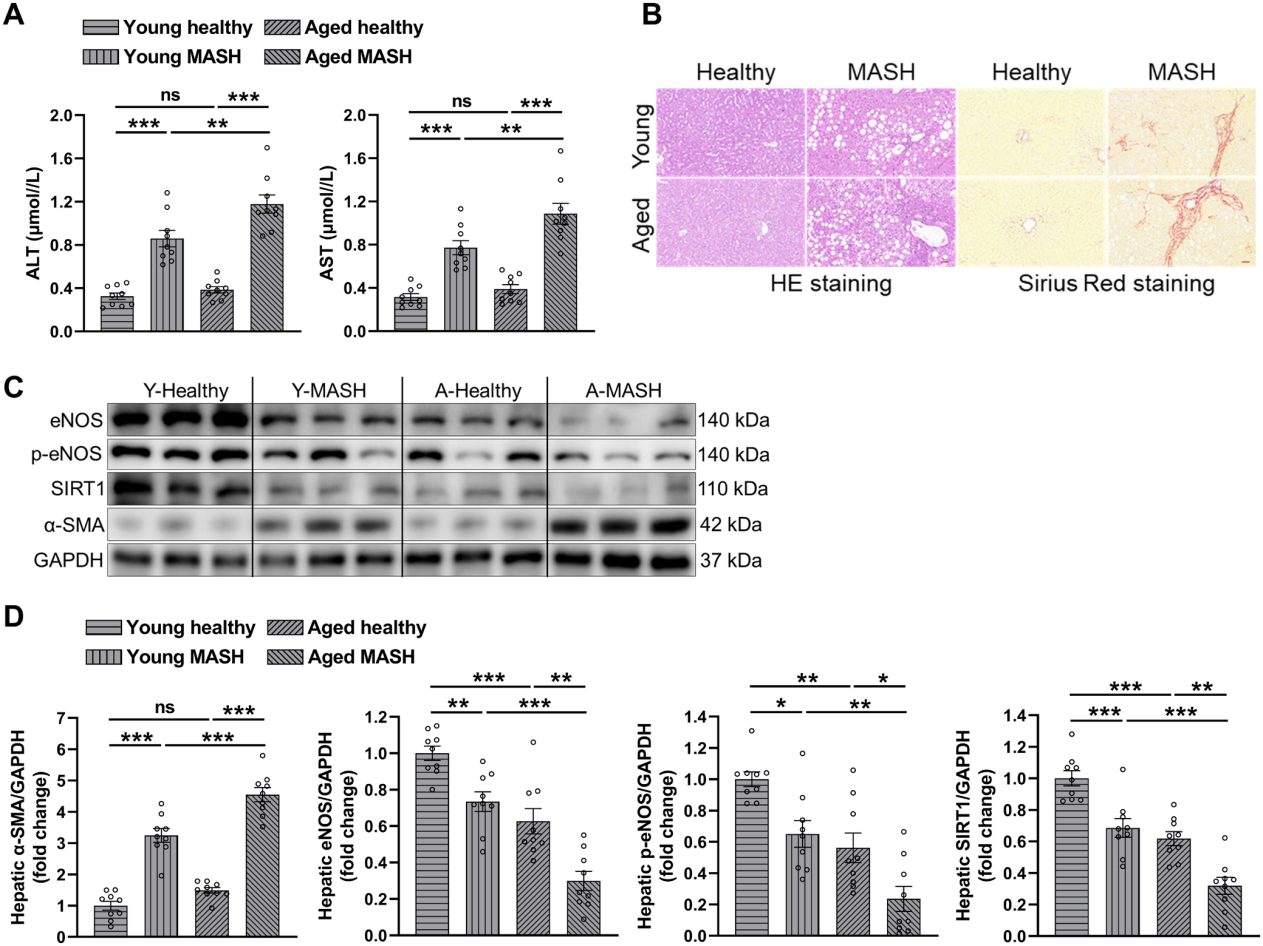
Effect of aging on liver injury, fibrosis, and LSECs‐related protein expression in MASH patients. (A) Serum ALT and AST levels. (B) Representative images of H & E staining and Sirius Red staining (scale bar = 50 μm). (C) Western blot and (D) quantitative analysis of hepatic eNOS, p‐eNOS, SIRT1, and α‐SMA protein expression levels. Data expressed as mean ± SEM (*N* = 9), one‐way ANOVA followed by Tukey's post hoc test, *p < 0.05, **p < 0.01, ***p < 0.001, and ns = not significant.

We next validated the results from animal experiments using liver samples from young and aged healthy controls and MASH patients. Serum ALT and AST levels were significantly elevated in MASH patients and were at highest levels in aged MASH patients (Figure 4A, details in Table S2). Accordingly, hematoxylin and eosin (HE) and Sirius Red staining indicated more severe liver injury and fibrosis in aged MASH patients (Figure 4B). Moreover, hepatic α‐SMA protein expressions were also remarkably increased in MASH patients and was highest in aged MASH patients (Figure 4C,D). Besides, we found that the protein expression levels of eNOS, p‐eNOS, and SIRT1 were significantly decreased in MASH livers and were at lowest levels in aged MASH patients (Figure 4C,D) suggesting that aged MASH patients predispose more severe LSECs dysfunction, liver injury, and fibrosis with accompanied decrement in hepatic SIRT1 levels in accordance with the observations from rodent MASH model.

### 2.5 Aging Aggravates Portal Venous Microcirculatory Dysfunction in MASH Liver

**FIGURE 5:**
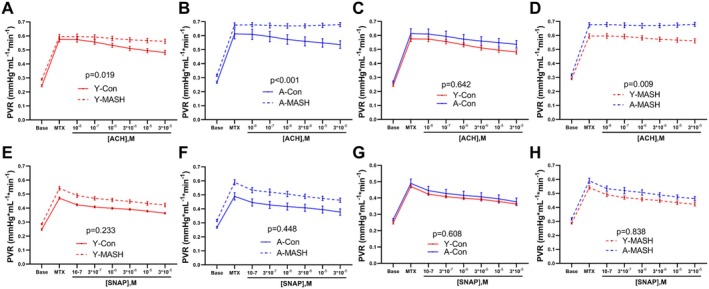
Effects of aging and MASH on the portal vascular bed reactivity. The PVR in response to increasing concentrations of (A–D) ACH and (E–H) SNAP of young (8 weeks) and aged (78 weeks) rats after 12 weeks of MCD diet or standard chow diet feeding. Data expressed as mean ± SEM (*N* = 9–12), general linear model for repeated measurements. ACH, acetylcholine; MTX, methoxamine; PVR, portal venous resistance; SNAP, *S*‐Nitroso‐*N*‐acetyl‐DL‐penicillamine.

The PVR at increasing concentrations of acetylcholine (ACH) were significantly higher in both young and aged MASH rats than in the corresponding controls (Figure 5A,B). In contrast, PVR at all concentrations of ACH did not alter between young control (Y‐Con) and A‐Con rats (Figure 5C), while were notably higher in A‐MASH rats than Y‐MASH rats (Figure 5D). However, there were no significant differences in PVR at increasing concentrations of SNAP when MASH rats were compared to the age‐matched control rats (Figure 5E,F). Likewise, aging had no effect on PVR at all concentrations of SNAP in the control and MASH rats (Figure 5G,H).

The relative PVR changes were remarkably lower in MASH rats than in control rats at all ACH concentrations (Figure S8A,B). In addition, relative PVR changes in control rats were not significantly different at all concentrations of ACH (Figure S8C), but was notably lower in A‐MASH rats than Y‐MASH rats (Figure S8D). The relative PVR change at all SNAP concentrations was comparable between the control and MASH groups in both young and aged rats (Figure S8E,F), and the intergroup comparisons also showed no differences for control and MASH rats (Figure S8G,H). These results suggest that microcirculatory dysfunction in the portal venous vascular bed in MASH rats while aging exacerbates disease‐associated endothelial dysfunction.

### 2.6 Hepatic Arterial Microcirculatory Dysfunction in MASH Liver

**FIGURE 6:**
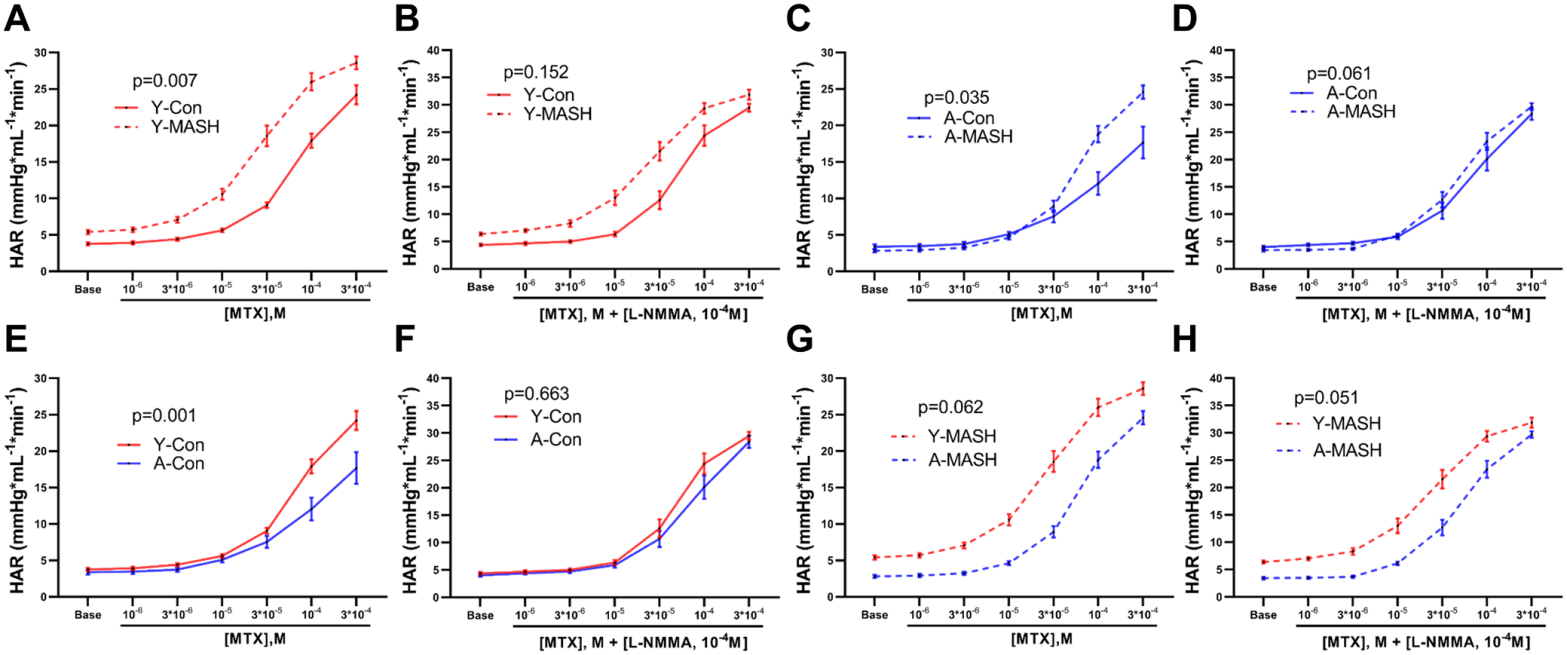
Influence of aging and MASH on the hepatic artery vascular bed reactivity. The HAR in response to increasing concentrations of MTX in the (A, C, E, and G) absence and (B, D, F, and H) presence of L‐NMMA of young (8 weeks) and aged (78 weeks) rats fed with standard chow diet or MCD diet. Data expressed as mean ± SEM (*N* = 9–12), general linear model for repeated measurements. HAR, hepatic arterial resistance; L‐NMMA, NG‐Methyl‐L‐arginine acetate salt; MTX, methoxamine.

Y‐MASH rats showed higher HAR and sensitivity to methoxamine (MTX) (determined by logEC50; −4.578 ± 0.060 in Y‐MASH, −4.123 ± 0.087 in Y‐Con, *p* < 0.0001) than Y‐Con rats (Figure 6A). NG‐Methyl‐L‐arginine acetate salt (L‐NMMA) supplementation eliminated the difference in HAR between the two groups, but different sensitivity to MTX remained (logEC50: −4.618 ± 0.064 in Y‐MASH, −4.301 ± 0.062 in Y‐Con, *p* = 0.0020, Figure 6B). The HAR was markedly increased in A‐MASH rats compared to A‐Con rats (Figure 6C), whereas the difference disappeared in the presence of L‐NMMA (Figure 6D). No differences were observed in the sensitivity to MTX between A‐Con and A‐MASH rats in the absence (logEC50: −3.718 ± 0.755 in A‐Con, −4.196 ± 0.073 in A‐MASH, *p* = 0.1791) or presence (logEC50: −4.058 ± 0.142 in A‐Con, −4.268 ± 0.067 in A‐MASH, *p* = 0.1562) of L‐NMMA (Figure 6C,D).

Intriguingly, HAR was considerably higher in Y‐Con rats than A‐Con rats (Figure 6E), whereas L‐NMMA erased the difference between the two groups (Figure 6F). Likewise, there were no significant differences in hepatic artery sensitivity to MTX between the two groups in the absence (logEC50: −4.123 ± 0.087 in Y‐Con, −3.718 ± 0.755 in A‐Con, *p* = 0.2926) or presence (logEC50: −4.301 ± 0.062 in Y‐Con, −4.058 ± 0.142 in A‐Con, *p* = 0.0578) of L‐NMMA (Figure 6E,F).

Moreover, Y‐MASH rats depicted a strong trend of higher HAR than A‐MASH rats both without and with L‐NMMA (Figure 6G,H). Furthermore, hepatic artery sensitivity to MTX in Y‐MASH rats was noticeably higher than A‐MASH rats without (logEC50: −4.578 ± 0.060 in Y‐MASH, −4.196 ± 0.073 in A‐MASH, *p* = 0.0005) and with (logEC50: −4.618 ± 0.064 in Y‐MASH, −4.268 ± 0.067 in A‐MASH, *p* = 0.0010) L‐NMMA (Figure 6G,H).

In addition, although the logEC50 in each group was not significantly different before and after the addition of L‐NMMA, the ΔlogEC50 values (logEC50 value without L‐NMMA − logEC50 value with L‐NMMA) were higher in the control group than in the MASH group (0.178 for Y‐Con and 0.34 for A‐Con, 0.04 for Y‐MASH and 0.072 for A‐MASH). The low ΔlogEC50 values and elevated HAR point to lower NO bioavailability and hepatic arterial microcirculatory dysfunction in MASH rats.

### 2.7 Increased SVR in A‐MASH Liver

**FIGURE 7:**
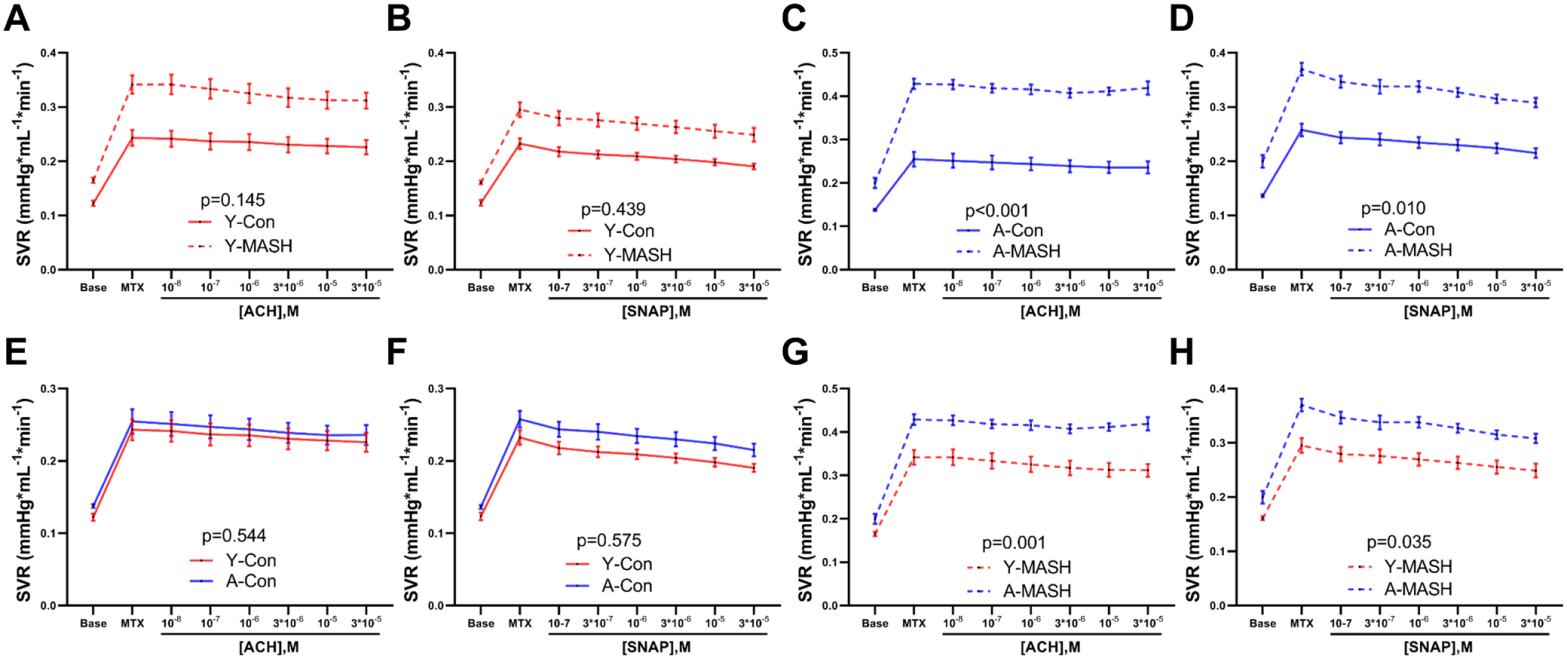
Combined effects of aging and MASH on the sinusoidal vascular bed reactivity in response to vasoactive drugs. The SVR in response to increasing concentrations of (A, C, E, and G) ACH and (B, D, F, and H) SNAP administration in the portal vein of young (8 weeks) and aged (78 weeks) control and MASH rats. Data expressed as mean ± SEM (*N* = 9–12), general linear model for repeated measurements. ACH, acetylcholine; MTX, methoxamine; SNAP, *S*‐Nitroso‐*N*‐acetyl‐DL‐penicillamine; SVR, sinusoidal vascular resistance.

The SVR did not change between the Y‐Con and Y‐MASH rats with increasing concentrations of ACH and SNAP (Figure 7A,B); however, aging resulted in a remarkable increment in SVR of MASH rats as compared to the control rats in the presence of ACH or SNAP (Figure 7C,D). Interestingly, no age‐associated effects on SVR were observed in control rats at all concentrations of ACH and SNAP (Figure 7E,F) implying that aging aggravates the SVR only in MASH rats. Likewise, A‐MASH rats had significantly higher SVR than Y‐MASH rats in the presence of both ACH and SNAP (Figure 7G,H).

In a similar manner, the relative SVR changes did not alter between the Y‐Con and Y‐MASH groups at all concentrations of ACH and SNAP (Figure S9A,B). However, the relative SVR change was significantly lower in the A‐MASH group than in the A‐Con group in the presence of ACH (Figure S9C), but the difference was eliminated by SNAP supplementation (Figure S9D). Besides, aging did not affect relative SVR changes in both control and MASH rats at all concentrations of ACH (Figure S9E,G) and SNAP (Figure S9F,H).

## 3 Discussion

Aging has been reported to increase the susceptibility of various liver diseases, such as alcoholic liver disease, liver fibrosis, and acute liver injury (Giavarotti et al. 2002; Maeso‐Diaz and Gracia‐Sancho 2020; Pinto et al. 2020; Ramirez et al. 2017; Ren et al. 2021). Previous studies predominantly focused on hepatocytes and HSCs (Ramirez et al. 2017; Rohn et al. 2020; Saito, Morine, and Shimada 2017), sparse research was conducted on changes in LSECs functioning and hepatic hemodynamics. In the present study, we observed that senescence compromised endothelial function and its protective ability against lipid accumulation in hepatocytes in vitro. Resveratrol treatment not only improved endothelial function but also partially recovered its protective effect against hepatocyte steatosis. In addition, animal experiments demonstrated more pronounced liver injury, steatosis, and fibrosis in A‐MASH rats than in Y‐MASH rats. Furthermore, endothelial and microcirculatory dysfunction were also more apparent in A‐MASH rats. These results were also obtained and validated through the examination of human liver samples from both young and aged MASH patients.

LSECs dysfunction has been reported previously to promote liver steatosis due to decreased eNOS‐derived NO (Garcia‐Villafranca, Guillen, and Castro 2003; Schild et al. 2008; Tateya et al. 2011). In this study, we found that young TSEC cells attenuated lipid accumulation in co‐cultured FFA‐treated AML12 cells, while this effect was abolished by L‐NAME supplementation. Besides, SNAP treatment reduced lipid accumulation in fat‐laden AML12 cells, implicating the crucial role of NO in lipid metabolism in hepatocytes. On the other hand, NO‐mediated protection against hepatocyte steatosis was compromised in co‐culture with senescent TSEC cells, inferring that senescence and aging‐associated endothelial dysfunction could impair fatty acid metabolism and lead to steatosis in adjacent hepatocytes. Furthermore, we were able to validate the above findings in rat primary hepatocytes and LSECs isolated from young and aged rats, proposing the crucial role of LSECs in hepatocyte lipid metabolism.

SIRT1 has garnered considerable attention for orchestrating various cellular processes to maintain genomic stability and overall cellular health (Grootaert and Bennett 2022; Lee et al. 2019). Luo et al. (2021) observed downregulated SIRT1 expression and capillarization in prematurely senescent LSECs in rat liver fibrosis model, while SIRT1 overexpression attenuated LSECs senescence and capillarization. However, the authors did not evaluate the effects of SIRT1 in regulating eNOS, p‐eNOS, or NO generation during premature LSECs senescence. Besides, Donato et al. detected remarkably reduced SIRT1 and p‐eNOS expressions in aortic endothelial cells from aged mice and aged healthy humans (Donato et al. 2011). Similarly, we observed significantly decreased levels of SIRT1 in senescent TSEC cells, accompanied by endothelial dysfunction delineated by markedly reduced eNOS, p‐eNOS, and intracellular NO levels. However, resveratrol treatment not only improved senescence‐associated endothelial dysfunction but also partially reinstated the protection of senescent endothelial cells against hepatocyte fat processing. Likewise, the protective effect of endothelial cells on hepatocyte steatosis was considerably diminished in primary LSECs isolated from aged rats, but was partially recovered by resveratrol treatment. These results robustly implicate the SIRT1 reduction in senescence‐associated endothelial dysfunction.

In animal experiments, A‐MASH rats suffered more severe liver injury, steatosis, and fibrosis than Y‐MASH rats. Considering the important role of eNOS‐derived NO in inhibiting hepatocyte steatosis and HSCs activation (Deleve, Wang, and Guo 2008; Garcia‐Villafranca, Guillen, and Castro 2003), the greatly decreased hepatic p‐eNOS protein levels in A‐MASH rats might cause hepatocyte steatosis and HSCs activation, ultimately exacerbating liver steatosis and fibrosis. Likewise, aging significantly reduced hepatic SIRT1 protein expression in normal rats. A‐MASH rats also appeared to have reduced hepatic SIRT1 protein expression but did not reach statistical significance. Furthermore, we observed age‐ and MASH‐related reduction in eNOS, p‐eNOS, and SIRT1 protein levels in human liver samples with more severe and pronounced liver injury and fibrosis in aged MASH patients than in young patients. Since our results confirmed an age‐ and disease‐associated regulation of SIRT1 with accompanied LSECs dysfunction; therefore, future studies aimed at endothelial cell‐specific SIRT1 expression will help to elucidate the SIRT1 contribution in aging‐ and disease‐associated LSECs dysfunction and its involvement in liver steatosis and fibrosis.

In pressure measurement, MASH rats showed endothelial dysfunction manifested by increased PVR and smaller relative PVR changes in response to ACH and unaltered responses to SNAP. Moreover, aging exacerbated portal venous microcirculation dysfunction in MASH rats but did not impact the control rats, consistent with previous report (Maeso‐Diaz et al. 2018). This suggests that age‐associated downregulation of hepatic p‐eNOS does not spontaneously lead to disease‐related phenotypes in healthy aged individuals, yet aggravates liver injury and disease progression in the presence of chronic liver disease. MASH rats showed higher HAR to MTX than control rats, but L‐NMMA eliminated this difference. Although HAR was significantly higher in Y‐MASH rats than in weight‐matched rats regardless of the presence or absence of L‐NMMA (Figure S10A,B), the addition of L‐NMMA erased the differences in sensitivity to MTX between the two groups with lower bioavailability of NO in MASH rats as depicted by logEC50 values. Besides, Y‐MASH rats have higher PVR and lower relative PVR change to ACH than weight‐match rats, and the response to SNAP was comparable (Figure S10C–F). These observations point to microcirculatory and endothelial dysfunction in MASH rats.

Portal venous resistance was significantly higher in Y‐MASH rats than in Y‐Con rats with increasing ACH concentrations (Figure 5A). However, SVR were similar between the two groups (Figure 7A). The hepatic arterial buffer response is an intrinsically regulated mechanism in which hepatic arterial flow changes inversely in response to alterations in portal venous flow to maintain stable total hepatic blood flow (Lautt 1983). Total hepatic blood flow and changes in hepatic arterial flow correlates with SVR according to Ohm's law (Zipprich, Loureiro‐Silva, D'Silva, and Groszmann 2008). Therefore, we conjecture that hepatic arterial buffer response reduces HAR in response to elevated PVR to maintain stable SVR values in Y‐MASH rats. We next analyzed the changes in HAR during ACH administration. Although, there was no significant difference in HAR, strong downward trends were observed in Y‐MASH rats compared to Y‐Con rats (Figure S11A). The absolute PVR (Figure 5B) and SVR values (Figure 7C) were both significantly higher in A‐MASH rats than in A‐Con rats during ACH administration, while the HAR were comparable between the two groups (Figure S11B). This could be attributed to the compromised hepatic arterial buffer response due to the hepatic arterial microcirculatory dysfunction in A‐MASH rats.

The spatial proximity between LSECs and HSCs brings growing interest in LSECs in liver fibrosis. Several studies demonstrated that LSECs dysfunction not only precedes liver fibrosis but also facilitates its progression in MASLD/MASH (Francque et al. 2012; Pasarin et al. 2012), and reinstatement of endothelial function could alleviate HSCs activation and liver fibrosis (Wang et al. 2013; Xie et al. 2012). Xie et al. (2012) found that activator of NO receptor soluble guanylate cyclase restored LSECs phenotype and promoted HSCs quiescence and regression of fibrosis in vivo. In our study, with the progression of MASH, aged rats showed more evident reduction of p‐eNOS protein and more severe endothelial dysfunction and microcirculatory impairment than young rats. Concurrently, HSCs activation and liver fibrosis were more severe in A‐MASH rats. These results implied that aging‐associated LSECs dysfunction was involved in aggravated liver injury and fibrosis in A‐MASH rats.

To the best of our knowledge, our study is the first one to comprehensively describe the age‐associated effects on MASH‐related liver fibrosis and hepatic hemodynamics, including basal pressures of the portal vein, hepatic artery, and hepatic sinusoids, along with pressure changes and mutual interactions in the presence of vasoactive drugs. The present study indicates the involvement of the SIRT1/p‐eNOS/NO pathway in aggravated MASLD caused by aging‐related LSECs dysfunction. Decreased SIRT1 and LSECs dysfunction could be potential therapeutic targets for mitigating the MASLD progression, but the underlying mechanism regarding the decreased SIRT1 in senescent and aged LSECs deserve further exploration.

## 4 Methods

### 4.1 In Vitro Endothelial Cell Senescence Model

Mouse liver sinusoidal endothelial cell line TSEC cells were cultured in DMEM/F12 medium supplemented with 5% FBS, 100 IU/mL penicillin/streptomycin, and 15 μg/mL ECGS. To develop a senescent cell model, TSEC cells were treated with different concentrations (0.1, 0.25, 0.5 and 1 μM) of etoposide (MedChemExpress, HY‐13629) for 2 days to induce DNA‐damage associated senescence. In the control groups, the cells were either treated with etoposide solvent DMSO or DMSO‐free normal culture media. Later, TSEC cells were washed with PBS and cultured in fresh media for additional 3 days.

### 4.2 Resveratrol and 1400 W Treatments

TSEC cells were treated with various concentrations (1, 2, 5, 10, 20, and 50 μM) of SIRT1 activator resveratrol (MedChemExpress, HY‐16561). Optimal resveratrol dosage was selected in accordance with cell viability after resveratrol treatment. Afterwards, to determine eNOS‐related endothelial cell functioning exclusively, the cells were treated with 20 μM of iNOS inhibitor 1400 W (MedChemExpress, HY‐18731) during the 2 days of etoposide treatment, while etoposide/1400 W enriched media was replaced with fresh resveratrol supplemented media in the following 3 days. The control groups contained DMSO or DMSO‐free culturing media as mentioned above.

### 4.3 Cell Viability Assay

The cell viability of TSEC cells after etoposide or resveratrol treatment was determined using Cell counting kit 8 (Abcam, ab228554) according to the manufacturer's instruction.

### 4.4 Senescence‐Associated β‐Galactosidase Staining

To have a uniform distribution, etoposide‐treated TSEC cells were re‐seeded in 6‐well plates 24 h prior to staining at different densities, e.g., the groups without etoposide or very low etoposide concentration were seeded with least number of cells while more cells were seeded in the treatment groups with increasing etoposide concentrations. After washing and fixation with 10% formalin, freshly prepared SABG staining solution was added to each well and incubated for 8 h in a 37°C incubator without CO_2_. Following washing with cold PBS, the cells were covered with cold PBS and captured under an inverted light microscope (Zeiss, Primo Vert).

### 4.5 Nitric Oxide Measurement

The intracellular nitrite concentrations were quantified by NO Assay Kit (Abcam, ab272517) to access intracellular NO levels according to the kit's manual. Subsequently, the measured nitrite concentration was normalized to the total cellular protein concentration (Thermo Fisher Scientific, 23225).

### 4.6 In Vitro Hepatocyte Steatosis Model

Mouse hepatocyte cell line AML12 cells were cultured in DMEM/F12 medium supplemented with 10% FBS, 100 IU/mL penicillin/streptomycin, 1× ITS, and 100 nM dexamethasone. To induce hepatocyte steatosis, AML12 cells were starved in serum‐free media for 12 h and then cultured in normal culturing media supplemented with low and high concentrations of FFA‐bovine serum albumin complex [palmitic acid (PA) 250 μM/oleic acid (OA) 500 μM and PA 500 μM/OA 1000 μM] for additional 24 h.

### 4.7 Oil Red O Staining

Free fatty acid‐treated AML12 cells and primary rat hepatocytes were washed and fixed with 10% formalin. Following fixation and washing steps, 60% isopropanol was added to the cells. Afterwards, isopropanol was removed and the cells were stained with Oil Red O working solution for 10 min. Later, the cells were washed and stained with hematoxylin. Subsequently, the cells were washed with water and were covered with water and captured under an inverted light microscope (Zeiss, Primo Vert).

### 4.8 Triglyceride Concentration Measurement

Intracellular triglyceride concentrations of steatotic AML12 cells, primary rat hepatocytes, and hepatic tissue from control and MASH rats were measured using triglyceride colorimetric assay kit (Elabscience, E‐BC‐K261‐M) following the manufacturer's instructions.

### 4.9 Co‐Culture of AML12 and TSEC Cells

To mimic the endothelial cell and hepatocyte interaction, in vitro co‐culture model was developed. AML12 cells were seeded in 6‐well transwell inserts at a density of 7.5 × 10^4^ cells/insert and steatosis was induced. Later, the inserts containing steatotic AML12 cells were transferred to the culture plates with senescent or non‐senescent TSEC cells and incubated for 24 h. Furthermore, to examine the effect of NO in hepatocyte steatosis, 3 mM eNOS inhibitor L‐NAME (MedChemExpress, HY‐18729A) (Deleve, Wang, and Guo 2008), or 100 μM NO donor SNAP (MedChemExpress, HY‐121526) (Kim et al. 2014) were added to the co‐culture media.

### 4.10 Animal Experiments

Young (8 weeks) and aged (78 weeks) male Wistar rats (Envigo RMS LLC) were housed in the humidity‐, temperature‐, and light/dark cycle‐controlled animal facility of Jena University Hospital and had free access to standard laboratory chow or MCD diet and water. The regional animal care committee of Thuringia approved all experimental procedures (License number: UKJ‐22‐009), and animal welfare was conducted in compliance with the ‘Animal Research: Reporting of In Vivo Experiments (ARRIVE)’ guidelines and current European Union regulations and the Protection of Animals Act. The rats were fed with MCD diet (ssniff Spezialdiäten GmbH, E15653‐94) for 12 weeks to induce MASH. Body weight and overall condition were monitored weekly during the experiment.

In situ bivascular liver perfusion was performed and hepatic hemodynamics and vascular resistance were determined as described previously (details in the Data S1) (Zipprich, Loureiro‐Silva, Jain, et al. 2008). Briefly, the rat was anesthetized with ketamine and xylazine, then a 14‐gauge catheter was inserted and secured in the portal vein and subsequently perfused with Krebs–Henseleit solution at 32 mL/min. The inferior vena cava was cut as an outflow tract. An 18‐gauge catheter was inserted and secured in the aorta, and the branches except the hepatic artery were ligated and subsequently perfused with Krebs–Henseleit solution at a rate of 8 mL/min. A polyethylene‐60 catheter was inserted from the right atrium through the caudal vena cava into the left hepatic lobe and secured. The perfusion pressure of the portal vein, hepatic artery, and sinusoid were measured constantly using three independent strain‐gauge transducers. Vascular resistance was calculated from the perfusion pressure and flow rate. Endothelial functions of the portal vein and hepatic artery were determined as the response to increasing doses of vasodilators and vasoconstrictors, respectively. Afterwards, liver and spleen samples were collected and weighed. In addition, vena cava blood and fresh tissue samples were collected from separate cohorts after perfusing the animals with cold PBS for 5 min at a rate of 5 mL/min to wash out blood.

### 4.11 Blood Biochemistry

Blood samples were collected from rats prior to bivascular liver perfusion and were referred to the clinical diagnostic laboratories at Jena University Hospital for biochemistry tests, including total protein, albumin, ALT, AST, total bilirubin, direct bilirubin, lactate dehydrogenase, alkaline phosphatase, γ‐glutamyltransferase (γ‐GT), triglycerides, cholesterol, cholinesterase, amylase, creatinine, urea, and glucose.

### 4.12 Primary Cell Isolation and Co‐Culture

Primary rat hepatocytes and LSECs were isolated from young (8 weeks) and aged (78 weeks) male Wistar rats as described previously (Zhang et al. 2016). Primary hepatocytes were cultured in DMEM (high glucose) supplemented with 10% FBS and 100 IU/mL penicillin/streptomycin. Primary LSECs were cultured in DMEM (low glucose) supplemented with 10% FBS, 100 IU/mL penicillin/streptomycin, and 10 ng/mL vascular endothelial growth factor. Primary hepatocytes were seeded in 24‐well plates at a density of 100,000 cells/cm^2^, and steatosis was induced as described above. Primary LSECs were seeded in collagen‐coated Transwell inserts at a density of 120,000 cells/cm^2^ with fresh resveratrol or DMSO supplemented media for 2 days. Later, the inserts containing primary LSECs were transferred to the culture plates with steatotic primary hepatocytes and incubated for 24 h. 3 mM L‐NAME or 100 μM SNAP was added to the co‐culture media (primary hepatocytes media as mentioned above) to examine the effect of NO in hepatocyte steatosis.

### 4.13 Clinical Samples Collection

Liver samples from male healthy controls and MASH patients were collected at the First Affiliated Hospital of Anhui Medical University between January 1, 2023, and April 30, 2024. All patients signed the informed consent form, and the study was approved by the Human Ethics Committee of the First Affiliated Hospital of Anhui Medical University (Approval number: Quick‐PJ‐2024‐04‐71). Individuals with age ranges of 18–35 and 55–70 years were defined as the young and aged groups, respectively. Healthy control liver tissue was obtained from patients who underwent hepatectomy for hepatic cyst or hemangioma (postoperative pathology confirmed the absence of liver steatosis and fibrosis). MASH liver tissue was obtained from patients who received hepatectomy for hepatic cyst or hemangioma without secondary liver steatosis etiology while MASH was confirmed by postoperative pathology.

### 4.14 Histology Stainings

Liver tissue samples were collected from a separate cohort of rats after perfusing with 1x cold PBS to wash out blood. Human and rat liver samples were fixed in 10% formalin solution, followed by H & E and Sirius Red staining according to the standard protocols. Finally, slices were observed and images were captured with a light microscope.

### 4.15 Hematoxylin and Eosin Staining

Human and rat liver samples were fixed in 10% formalin solution and processed with automatic tissue processor (Diapath, SDSDT9000/Leica, TP1020). Afterwards, the formalin fixed samples were embedded in paraffin and cut into 5 μm sections using a microtome (Leica, RM2016/RM2255). For histology, the slices were deparaffinized in xylene and rehydrated in descending ethanol solutions. Subsequently, the slides were stained with Hematoxylin and Eosin Y solutions, dehydrated, and cleared by immersing in ascending ethanol solutions and xylene, respectively. Finally, slices were mounted with mounting medium and images were captured with a light microscope (Zeiss, AxioObserver Z1/Nikon, ECLIPSE E100).

### 4.16 Sirius Red Staining

Liver slices were deparaffinized and rehydrated in xylene and descending ethanol solutions respectively. Slices were stained with Picro Sirius Red solution for 60 min, then quickly rinsed in 0.5% acetic acid solution. After dehydration and clearing, the slices were mounted and captured under a light microscope.

### 4.17 Western Blot

Cells or liver tissue was lysed in lysis buffer (9803, Cell Signaling Technology) supplemented with complete protease inhibitor (S8820, Sigma) and phosphatase inhibitors (4906837001, Sigma), followed by brief homogenization. Homogenate was centrifuged in pre‐cooled micro‐centrifuge, and supernatant was collected for protein concentration measurement by BCA assay. Samples containing equal amount of total protein were denatured in loading buffer for electrophoresis, then transferred to activated polyvinylidene difluoride (PVDF) membranes. After transfer, membranes were blocked in 5% milk at room temperature for 1 h with constant rocking and incubated in primary antibodies diluted in blocking solution overnight at 4°C with gentle rocking. The primary antibodies used are mentioned in Table S3. Membranes were washed with TBST and incubated in HRP‐conjugated secondary antibody (Table S3) diluted in blocking solution for 1 h at room temperature with gentle rocking. Finally, membranes were incubated in freshly prepared chemiluminescent HRP substrate (Millipore, WBKLS0500) and chemiluminescence signals were captured using the gel documentation system (Syngene, GBOX‐Chemi‐XX6).

### 4.18 Quantitative Real‐Time Polymerase Chain Reaction (RT‐PCR)

Total RNA was extracted from cells and liver tissues using the NucleoSpin RNA plus kit (Macherey‐Nagel, 740984.250) and converted into complementary DNA (cDNA) with high‐capacity cDNA reverse transcription kit (Applied Biosystems, 4368814). RT‐PCR was performed using Maxima SYBR Green qPCR master mix (Thermo Fisher Scientific, K0253). The relative mRNA levels were calculated by the 2^−∆∆Ct^ method and normalized to β‐actin. Primer sequences are mentioned in Table S4.

### 4.19 Statistical Analysis

All data are expressed as mean ± SEM and analyzed using GraphPad Prism 8.0 software. Differences between groups were analyzed using two‐tailed Student's *t*‐test or one‐way ANOVA, followed by Tukey's post hoc test. For pressure measurement, data was analyzed by general linear model for repeated measurements using SPSS Statistics 26.0. The sensitivity of hepatic artery to MTX was measured by logEC50 (van der Horst et al. 2022), and logEC50 values of concentration–responses curve were calculated by nonlinear regression analysis (four parameters) using GraphPad Prism 8.0 software. Statistical significances were expressed as */#*p* < 0.05, **/##*p* < 0.01, ***/###*p* < 0.001, and ns/*ns* = not significant.

## Author Contributions

Q.D. and Q.A. conceptualized and planned the experiments; Q.D. carried out the in vitro experiments and performed the bivascular liver perfusion; Q.A. M.R., and N.S. assisted in animal experiments; N.S. and M.R. helped with the qPCR; H.Z. assisted in clinical liver sample collection and staining; Q.D. and Q.A. analyzed the data and wrote the manuscript; A.Z. provided funding, helped with data analysis and interpretation, revised the manuscript, and supervised the whole study.

## Conflicts of Interest

The authors declare no conflicts of interest.

## Supporting information


Data S1.

## Data Availability

Data supporting the results of this study are available from the corresponding author upon reasonable request.
