# Supplementary material for: Aging‐Associated Liver Sinusoidal Endothelial Cells Dysfunction Aggravates the Progression of Metabolic Dysfunction‐Associated Steatotic Liver Disease
Source: Aging Cell. 2025 Feb 6;24(5):e14502. doi: 10.1111/acel.14502 (PMC12073894; doi:10.1111/acel.14502)
Supplement: Supplementary file 1 — Data S1. [file ACEL-24-e14502-s001.docx]

**In situ bivascular liver perfusion**

After anesthesia with ketamine (WDT, Bela-Pharm 1009408) and xylazine (WDT, Elanco 1003268), bivascular liver perfusion was performed. After opening the abdomen, loose ligatures were placed around the aorta cranial to the celiac artery, around the superior mesenteric artery immediately after branching from the aorta, and the aorta caudal to the mesenteric artery. Meanwhile, the buffers were continuously warmed during the whole experiment in water bath at 39 °C. Left gastric and splenic arteries were ligated at the origin from the celiac artery, and a lose ligature was placed around the esophagus. Left and right renal arteries and gastroduodenal artery were also ligated. The portal vein was cannulated with a 14-gauge catheter (BD Angiocath, 382268) and perfused with pre-warmed oxygenated Krebs–Henseleit solution (118 mM NaCl, 25 mM NaHCO_3_, 4.7 mM KCl, 1.2 mM KH_2_PO_4_, 1.2 mM MgSO_4_, 11.1 mM Dextrose, 2.4 mM CaCl_2_, 25 mM HEPES) at the rate of 32 mL/minute in a non-recirculating mode. The inferior vena cava was cut as outlet immediately. The aorta was cannulated with an 18-gauge catheter (BD Angiocath, 381147) and perfused with oxygenated Krebs–Henseleit solution at the rate of 8 mL/minute in a non-recirculating mode. The tip of the catheter was positioned close to the branch of the celiac artery. Afterwards, the ligatures around the superior mesenteric artery, the esophagus and all around the aorta were ligated. A 14-gauge catheter was inserted into the inferior vena cava and secured. To measure the sinusoidal pressure, thorax was opened and a polyethylene-60 catheter (Vetanimalab, BTPE-60) was guided from the right atrium, through the thoracic segment of the caudal vena cava into the left hepatic lobe and wedged in the hepatic vein. A ligature was placed and ligated around the thoracic segment of the inferior vena cava to secure the wedged catheter. The perfusion pressure of the portal vein and the hepatic artery were measured constantly using two independent strain-gauge transducers (Spectramed, P23XL) during the stabilization and the experimental period. In parallel, the sinusoidal pressure was measured using a third independent strain-gauge transducer during the experimental period. Initially, all pressure measurement systems were calibrated with the zero point at the level of the hepatic hilium before each experiment. All pressures were continuously recorded by Chart 5.5 program using MacLab / 4e hardware (AD instruments). During perfusion and subsequent pressure measurement, thin-walled O_2_/CO_2_ permeable silicone tubes were interposed between the peristaltic pump and the catheters of the portal vein and hepatic artery and placed in an O_2_-filled bottle to continuously oxygenate the perfusate.

The portal venous system was pre-constricted following the stabilization period by adding pre-established dose of 10^−4^ M methoxamine (MTX, Sigma, M6524) to the perfusate. Afterwards, dose-response vasodilatation curves to six increasing concentrations of vasodilator ACH (from 10^−8^ to 3×10^−5^ M, Sigma, A6625) and nitric oxide donor S-Nitroso-N-acetyl-DL-penicillamine (SNAP, from 10^−7^ to 3×10^−5^ M, HY-121526) were measured to determine the endothelial function. The sinusoidal pressure was determined by interrupting the wedged catheter outflow. In addition, the dose-response pressure curves of the hepatic artery were evaluated with addition of 6 consecutively increasing concentrations of vasoconstrictor MTX (10^-6^ to 3×10^-4^ M) in the perfusate in the presence or absence of nitric oxide synthase (NOS) inhibitor N^G^-Methyl-L-arginine acetate salt (L-NMMA, 10^-4^ M, Sigma, M7033).

Hepatic arterial vascular resistance (HAR), portal venous vascular resistance (PVR), and sinusoidal vascular resistance (SVR) were calculated from the perfusion pressure and flow rate (HAR = hepatic arterial pressure/hepatic arterial flow rate, PVR = portal venous pressure/portal venous flow rate, SVR = sinusoidal pressure/sum of portal venous and hepatic arterial flow rate).

**Supplementary table 1.** Blood biochemical tests of young and aged control and MASH rats.

| Test items | Y-Con | Y-MASH | A-Con | A-MASH | P1 | P2 | P3 | P4 |
| --- | --- | --- | --- | --- | --- | --- | --- | --- |
| Total protein (g/L) | 61.45±0.45 | 50.98±1.39 | 60.83±2.23 | 51.83±1.75 | 0.0021 | 0.0081 | 0.9944 | 0.9856 |
| Albumin (g/L) | 42.33±0.81 | 38.33±0.69 | 40.17±1.40 | 37.00±1.65 | 0.1746 | 0.3523 | 0.6582 | 0.8905 |
| ALT (µmol/L) | 0.58±0.10 | 1.38±0.13 | 0.66±0.12 | 1.99±0.13 | 0.0015 | <0.0001 | 0.9692 | 0.0169 |
| AST (µmol/L) | 1.26±0.16 | 2.23±0.19 | 1.21±0.17 | 2.97±0.25 | 0.0208 | <0.0001 | 0.9986 | 0.0998 |
| Total bilirubin (µmol/L) | <3* | 5.17±0.44 | <3* | 9.17±1.30 | / | / | / | 0.0238^#^ |
| Direct bilirubin (µmol/L) | <3* | 4.75±0.32 | <3* | 8.87±1.42 | / | / | / | 0.0272^#^ |
| Lactate dehydrogenase (µmol/L) | 3.26±0.33 | 6.79±0.50 | 3.44±0.74 | 8.58±1.41 | 0.0645 | 0.0046 | 0.9990 | 0.5389 |
| Alkaline phosphatase (µmol/L) | 1.49±0.17 | 1.69±0.14 | 1.70±0.15 | 1.78±0.11 | 0.8035 | 0.9860 | 0.7880 | 0.9822 |
| γ-glutamyltransferase (µmol/L) | <0.05* | <0.05* | <0.05* | <0.05* | / | / | / | / |
| Triglycerides (mmol/L) | 1.15±0.13 | 0.22±0.02 | 1.49±0.17 | 0.31±0.03 | 0.0001 | <0.0001 | 0.2051 | 0.9352 |
| Cholesterol (mmol/L) | 2.03±0.05 | 0.59±0.07 | 3.35±0.28 | 1.05±0.21 | 0.0002 | <0.0001 | 0.0006 | 0.3536 |
| Cholinesterase (µmol/L) | 3.67±0.56 | 5.50±0.77 | 5.83±0.89 | 6.33±0.61 | 0.3801 | 0.9694 | 0.2445 | 0.8775 |
| Amylase (µmol/L) | 35.27±2.29 | 22.75±1.07 | 30.00±1.28 | 24.67±2.44 | 0.0017 | 0.2852 | 0.2935 | 0.9094 |
| Creatinine (µmol/L) | 34.17±1.12 | 32.17±1.04 | 44.00±2.43 | 41.83±5.20 | 0.9717 | 0.9645 | 0.1752 | 0.1864 |
| Urea (mmol/L) | 6.72±0.47 | 7.13±0.44 | 6.02±0.33 | 5.95±0.48 | 0.9246 | 0.9996 | 0.7279 | 0.3209 |
| Glucose (mmol/L) | 13.87±0.62 | 7.42±0.49 | 12.55±0.70 | 7.30±0.34 | <0.0001 | <0.0001 | 0.4380 | 0.9991 |

Data expressed as mean ± SEM.

*Below the limit of detection.

Y-Con: Young control; Y-MASH: Young MASH; A-Con: Aged control; A-MASH: Aged MASH.

P1: Y-Con versus Y-MASH; P2: A-Con versus A-MASH; P3: Y-Con versus A-Con; P4: Y-MASH versus A-MASH.

**Supplementary table 2.** Clinical characteristics of young and aged healthy controls and MASH patients.

| Variables | Y-H (n=9) | Y-M (n=9) | A-H (n=9) | A-M (n=9) | P1 | P2 | P3 | P4 |
| --- | --- | --- | --- | --- | --- | --- | --- | --- |
| Age (years) | 24.67±2.62 | 26.89±2.42 | 59.56±3.30 | 58.78±3.56 | 0.464 | 0.955 | <0.001 | <0.001 |
| Body mass index (Kg/m^2^) | 22.63±1.76 | 26.58±2.12 | 23.29±1.32 | 26.96±1.63 | <0.001 | 0.001 | 0.873 | 0.972 |
| Disease duration* (years) | / | 10.89±2.18 | / | 13.44±2.95 | / | / | / | 0.066 |
| Total bilirubin (µmol/L) | 11.77±2.11 | 16.10±2.21 | 12.34±1.84 | 18.73±3.38 | 0.007 | <0.001 | 0.965 | 0.161 |
| ALT (µmol/L) | 0.33±0.09 | 0.86±0.22 | 0.39±0.08 | 1.18±0.24 | <0.001 | <0.001 | 0.899 | 0.004 |
| AST (µmol/L) | 0.32±0.09 | 0.77±0.18 | 0.39±0.12 | 1.09±0.27 | <0.001 | <0.001 | 0.851 | 0.007 |
| Alkaline phosphatase (µmol/L) | 0.93±0.22 | 1.12±0.25 | 0.95±0.20 | 1.29±0.34 | 0.450 | 0.057 | 0.998 | 0.548 |
| γ-glutamyltransferase (µmol/L) | 0.57±0.16 | 0.74±0.20 | 0.53±0.15 | 0.86±0.17 | 0.203 | 0.002 | 0.955 | 0.510 |
| Albumin (g/L) | 46.33±1.44 | 43.42±1.71 | 45.11±1.46 | 41.34±2.23 | 0.011 | <0.001 | 0.545 | 0.099 |

Data expressed as mean ± SEM.

Y-H: Heathy young; A-H: Healthy aged; Y-M: Young MASH patients; A-M: Aged MASH patients.

P1: Y-H versus Y-M; P2: A-H versus A-M; P3: Y-H versus A-H; P4: Y-M versus A-M.

*Time from first diagnosis of fatty liver to liver sample collection.

**Supplementary table 3.** List of antibodies used for Western Blot

| **Antibody** | **Dilution** | **Manufacturer** |
| --- | --- | --- |
| Anti-eNOS rabbit antibody | 1:2000 | R&D Systems, HAF008 |
| Anti-iNOS rabbit antibody | 1:2000 | R&D Systems, HAF007 |
| Anti-phospho-eNOS (Ser1177) rabbit antibody | 1:1000 | Invitrogen, PA1-037 |
| Anti-αSMA rabbit antibody | 1:1000 | Novus Biologicals, NB300-605 |
| Anti-GAPDH mouse antibody | 1:1000 | Invitrogen, MA5-14957 |
| β-Actin rabbit antibody (HRP conjugate) | 1:1000 | Abcam, ab5694 |
| Recombinant anti-SIRT1 rabbit antibody | 1:1000 | Proteintech, 60004-1-Ig |
| Recombinant anti-p21 rabbit antibody | 1:1000 | Cell Signaling Technology, 5125 |
| Anti-rabbit IgG antibody (HRP conjugate) | 1:1000 | Abcam, ab189494 |
| Anti-mouse IgG antibody (HRP conjugate) | 1:1000 | Abcam, ab109199 |

**Supplementary table 4.** List of primer sequences

| **Genes** | **Species** | **Forward primer (5` → 3`)** | **Reverse primer (5` → 3`)** |
| --- | --- | --- | --- |
| β-actin | Mouse | GGCTGTATTCCCCTCCATCG | CCAGTTGGTAACAATGCCATG |
| SIRT1 | Mouse | TGATTGGCACCGATCCTCG | CCACAGCGTCATATCATCCAG |
| eNOS | Mouse | GGCAACTTGAAGAGTGTGGG | CTGAGGGTGTCGTAGGTGATG |
| β-actin | Rat | CCCGCGAGTACAACCTTCTT | CGACGAGCGCAGCGATA |
| SIRT1 | Rat | TGAAGCTGTTCGTGGAGATATTTTT | CATGATGGCAAGTGGCTCAT |
| PPARα | Rat | CAATGCCCTCGAACTGGATG | ATCCCCTCCTGCAACTTCTC |
| SREBP1c | Rat | AGGAGGCCATCTTGTTGCTT | GTTTTGACCCTTAGGGCAGC |
| ACC | Rat | ACCTCAACCACTACGGCATGA | AGGTGGTGTGAAGGCGTTGT |


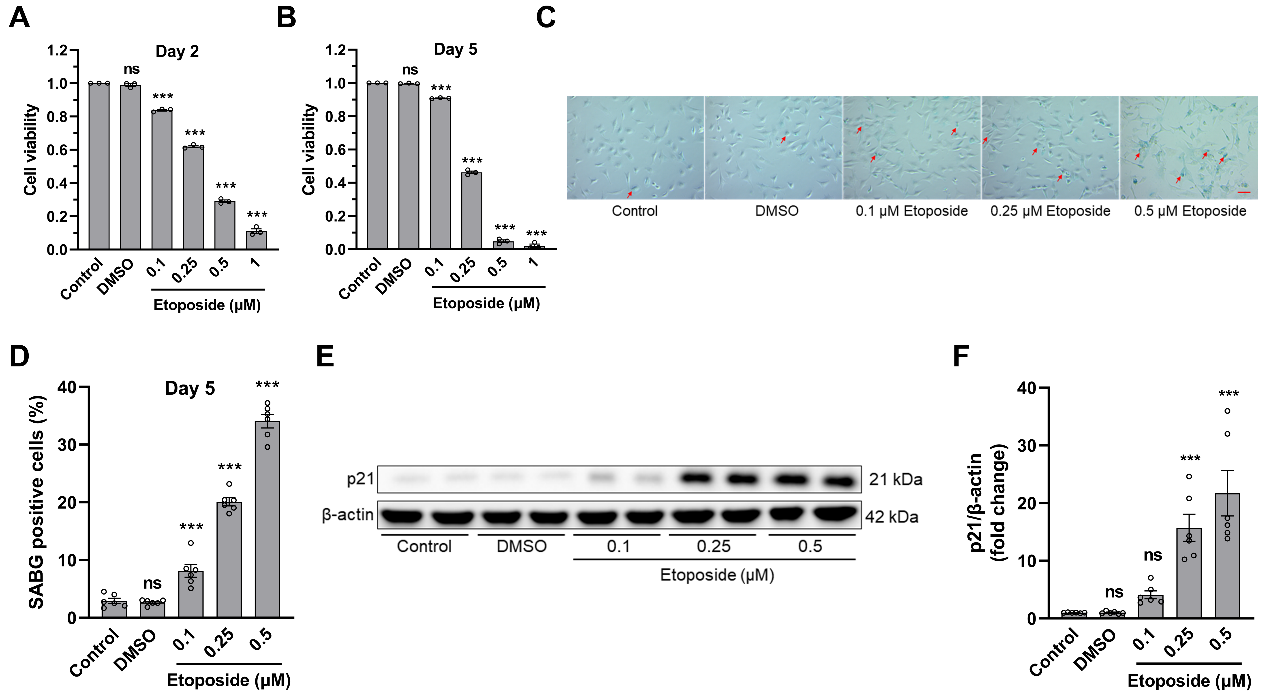


**Supplementary figure 1.** **Development of in vitro senescent endothelial cell model.** (A) Cell viability of TSEC cells at day 2 and (B) at day 5 after etoposide treatment to establish endothelial cell senescence model (N = 3). (C) Senescence-associated β galactosidase staining (SABG) staining (scale bar = 5 μm) and (D) quantitative analysis showing the percentage of senescent cells (SABG positive) at day 5 (N = 6). (E) Western blot and (F) quantitative analysis of p21 protein levels in senescent TSEC cells at day 5 (N = 6). Data expressed as mean ± SEM, one-way ANOVA followed by Tukey's post- hoc test. *P < 0.05, **P < 0.01, ***P < 0.001, and ns = not significant.


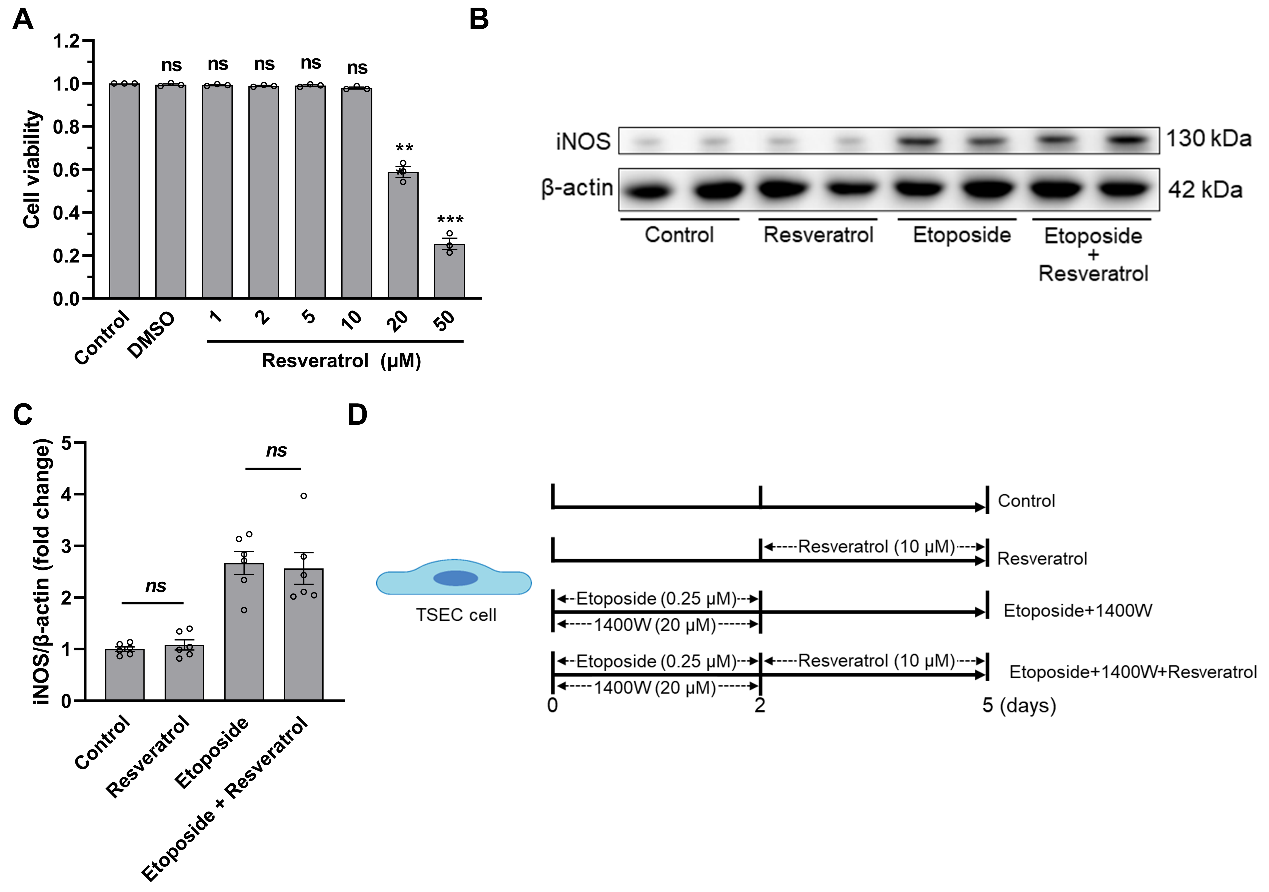


**Supplementary figure 2. Determination of optimal resveratrol concentration and experimental demonstration.** (A) Cell viability of TSEC cells after treatment with different concentration of resveratrol (N = 3,). (B) Western blot and (C) quantitative analysis of TSECs iNOS protein level after etoposide and/or resveratrol treatment (N = 6). (D) Demonstration of experimental set up and different treatment groups of TSEC cells. Data expressed as mean ± SEM, one-way ANOVA followed by Tukey's post- hoc test. In A, *** and ns show comparison vs control group; in C, *ns* show inter-group comparisons. *P < 0.05, **P < 0.01, ***P < 0.001, and ns/*ns* = not significant.


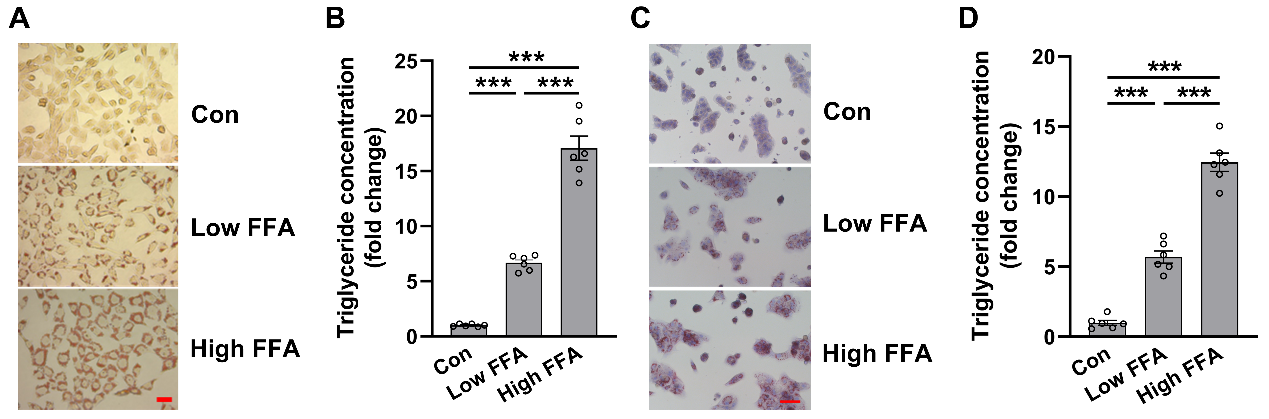


**Supplementary figure 3.** **Establishment of in vitro hepatic steatosis model.** (A) Oil Red O staining (scale bar = 5 μm) and (B) quantification of triglyceride concentration in AML12 cells treated with low (PA 250 µM/OA 500 µM), and high (PA 500 µM/OA 1000 µM) FFA concentration. (C) Oil Red O staining (scale bar = 100 μm) and (D) quantification of triglyceride concentration in primary rat hepatocytes treated with low and high FFA concentration. Data expressed as mean ± SEM (N = 6), one-way ANOVA followed by Tukey's post- hoc test. ***P < 0.001. FFA, free fatty acid; PA, palmitic acid; OA, oleic acid.


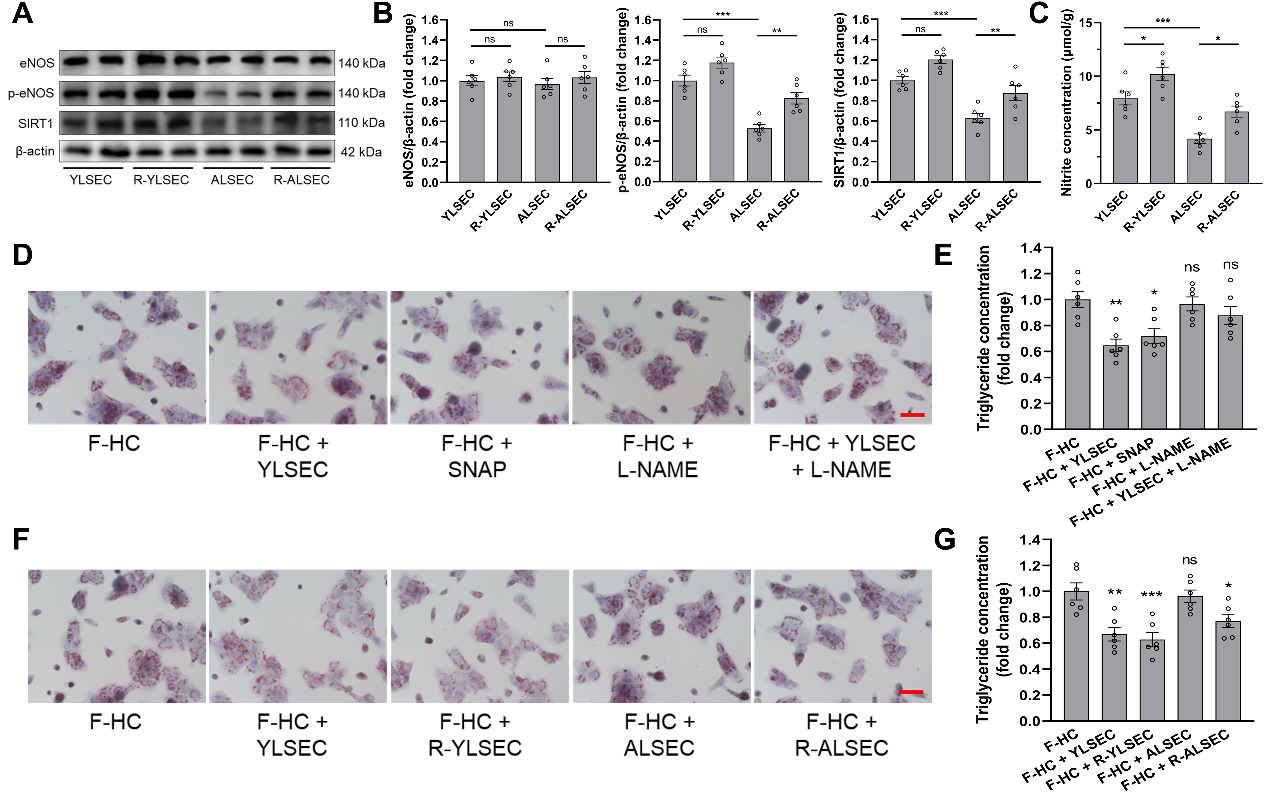


**Supplementary figure 4.** **The effects of primary rat LSECs on primary rat hepatocyte steatosis.** (A) Western blot, (B) quantitative analysis of eNOS, p-eNOS, and SIRT1 protein levels, and (C) nitrite levels of primary young and aged LSECs before and after resveratrol treatment. (D) Oil Red O staining and (E) triglyceride concentration quantification in F-HC mono-culture or co-cultured with YLSEC, mono-culture with addition of SNAP, or L-NAME, or co-cultured with Y-LSEC in the presence of L-NAME. (F) Oil Red O staining and (G) triglyceride concentration estimation in F-HC cultured alone or co-cultured with the indicated LSECs. In D and F, scale bar = 100 μm. Data expressed as mean ± SEM (N = 6), one-way ANOVA followed by Tukey's post- hoc test. In E and G, comparison vs control group. *P < 0.05, **P < 0.01, ***P < 0.001, and ns = not significant. F-HC, high free fatty acid-treated primary hepatocyte; YLSEC, young primary rat liver sinusoidal endothelial cells; R-YLSEC, resveratrol-treated young primary LSECs; ALSEC, aged primary LSECs; R-ALSEC, resveratrol-treated aged primary LSECs; SNAP, S-Nitroso-N-acetyl-DL-penicillamine; L-NAME, NG-Nitroarginine methyl ester hydrochloride.


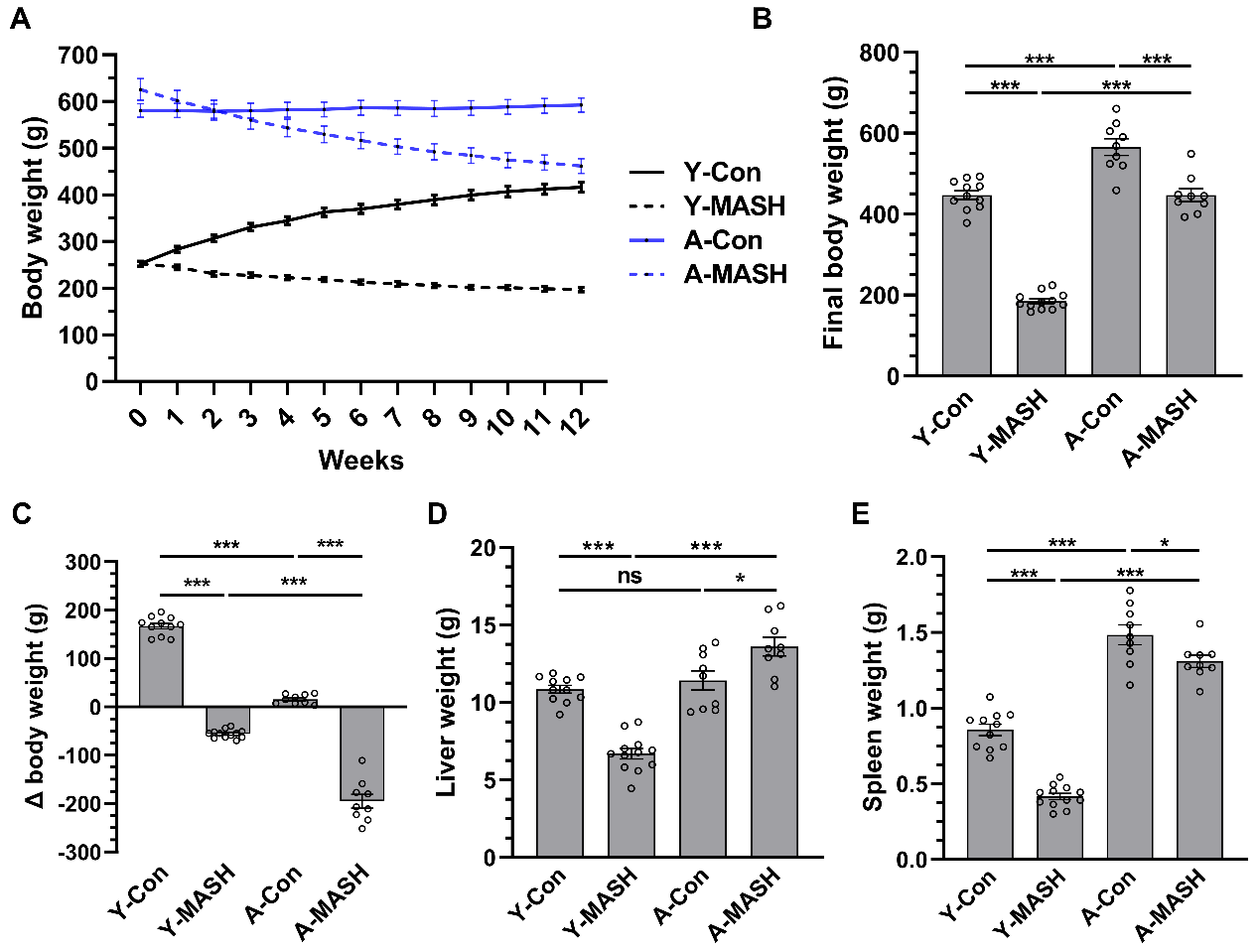


**Supplementary figure 5.** **Effect of MCD diet on different physiological parameters of rats.** (A) Body weight curve, (B) final body weight, (C) Δ body weight (weight at start of MCD diet – weight at end of MCD diet), (D) liver weight and (E) spleen weight of young (8 weeks) and aged (78 weeks) rats after feeding with standard chow diet or MCD diet for 12 weeks. Data expressed as mean ± SEM (N = 9-12), one-way ANOVA followed by Tukey's post- hoc test, *P < 0.05, **P < 0.01, ***P < 0.001, and ns = not significant.


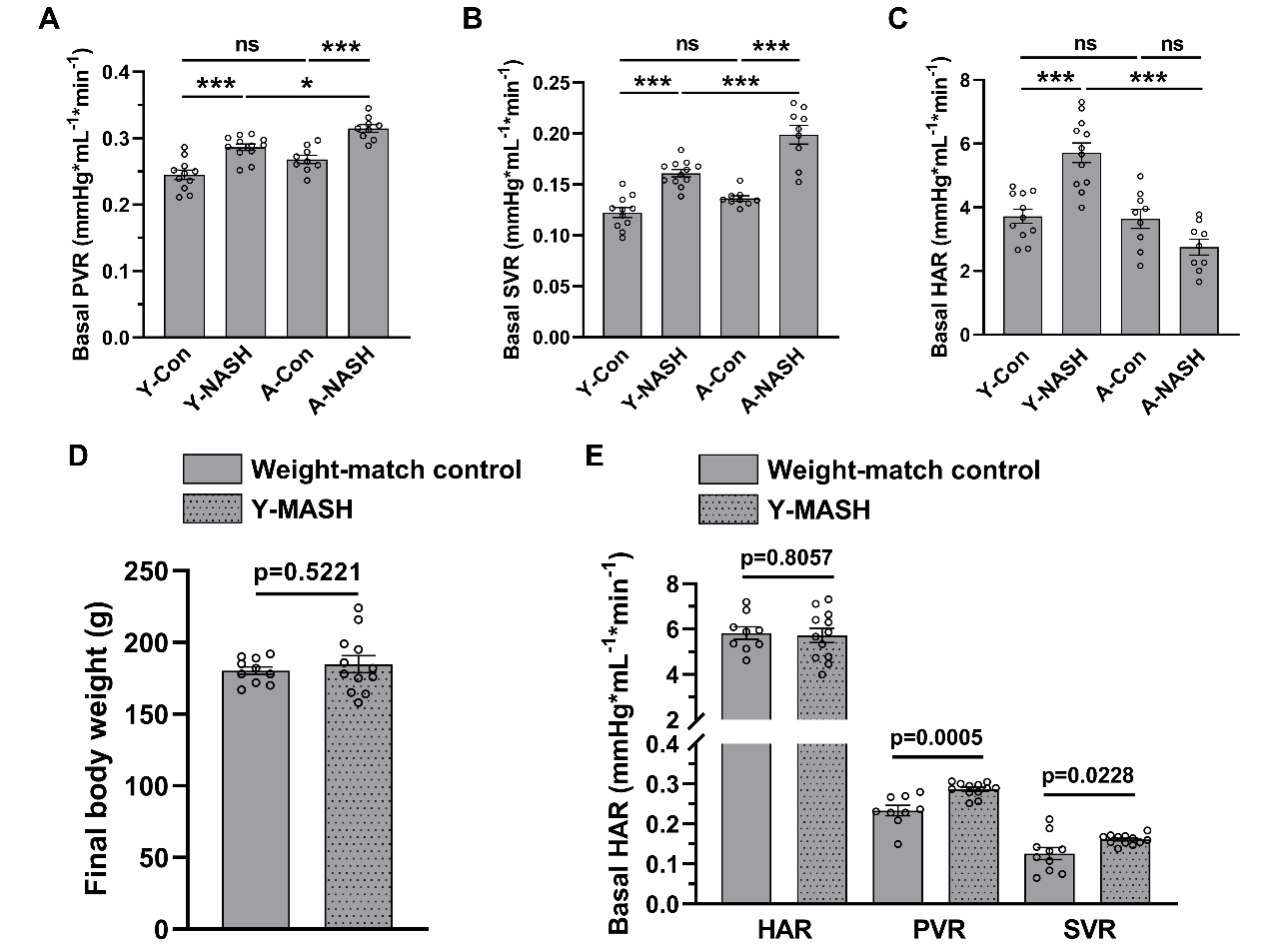


**Supplementary figure 6. Comparison of basal vascualr resistance.** (A-C) basal PVR, SVR, and HAR of young and aged control and MASH rats. (D) Final body weight and (E) basal HAR, PVR, and SVR of young (8 weeks) rats after 12 weeks of MCD diet consumption and weight-match control rats fed with standard chow diet. Data expressed as mean ± SEM (N = 9-12). For A-C, one-way ANOVA followed by Tukey's post- hoc test, *P < 0.05, **P < 0.01, ***P < 0.001, and ns = not significant. For D and E, two-tailed Student's t-test. HAR, hepatic arterial resistance; PVR, portal venous resistance; SVR, sinusoidal vascualr resistance.


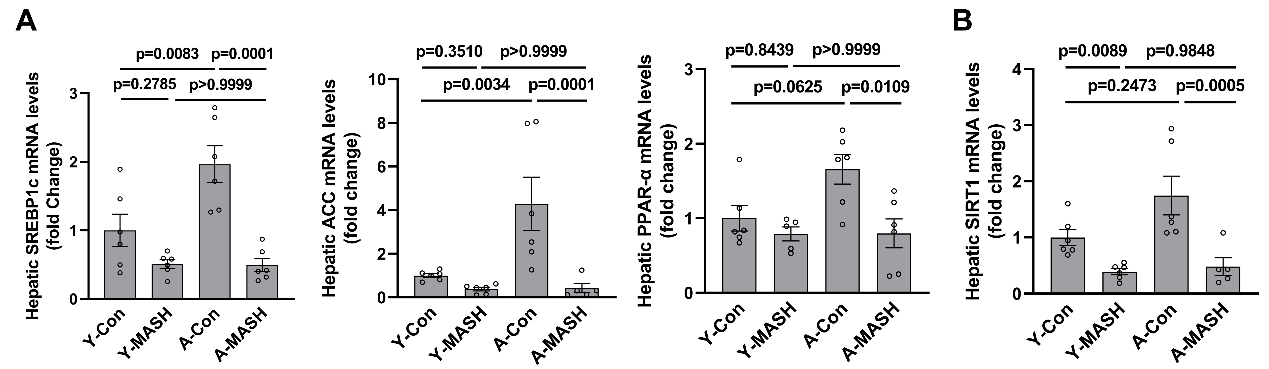


**Supplementary figure 7. Age- and MASH-associated changes in hepatic lipid metabolic genes and SIRT1 gene.** Quantitative analysis of hepatic mRNA expression levels of (A) SERBP1c, ACC and PPAR-α and (B) SIRT1 of young (8 weeks) and aged (78 weeks) rats after 12 weeks of MCD diet or standard chow diet feeding. Data expressed as mean ± SEM (N = 6), one-way ANOVA followed by Tukey's post- hoc test. SERBP1c, Sterol regulatory element binding protein 1c; ACC, Acetyl-CoA carboxylase; PPAR-α; Peroxisome proliferator-activated receptor alpha.


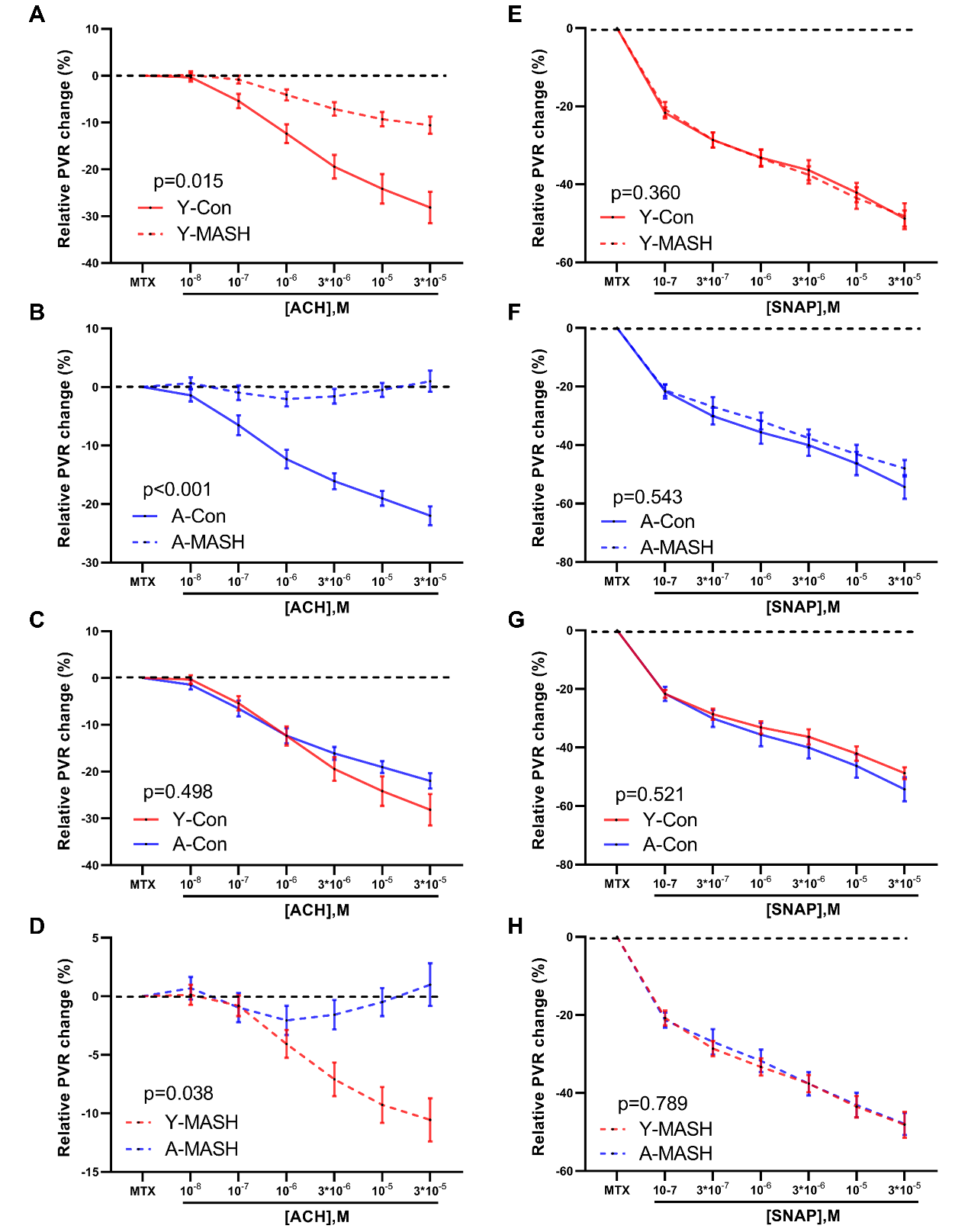


**Supplementary figure 8.** **Age and MASH -associated changes in the relative portal vascular bed reactivity.** The relative PVR change in response to increasing concentrations of (A, B, C, and D) ACH and (E, F, G, and H) SNAP of young (8 weeks) and aged (78 weeks) control and diet-induced MASH model. Data expressed as mean ± SEM (N = 9-12), general linear model for repeated measurements. PVR, portal venous resistance; MTX, methoxamine; ACH, acetylcholine; SNAP, S-Nitroso-N-acetyl-DL-penicillamine.


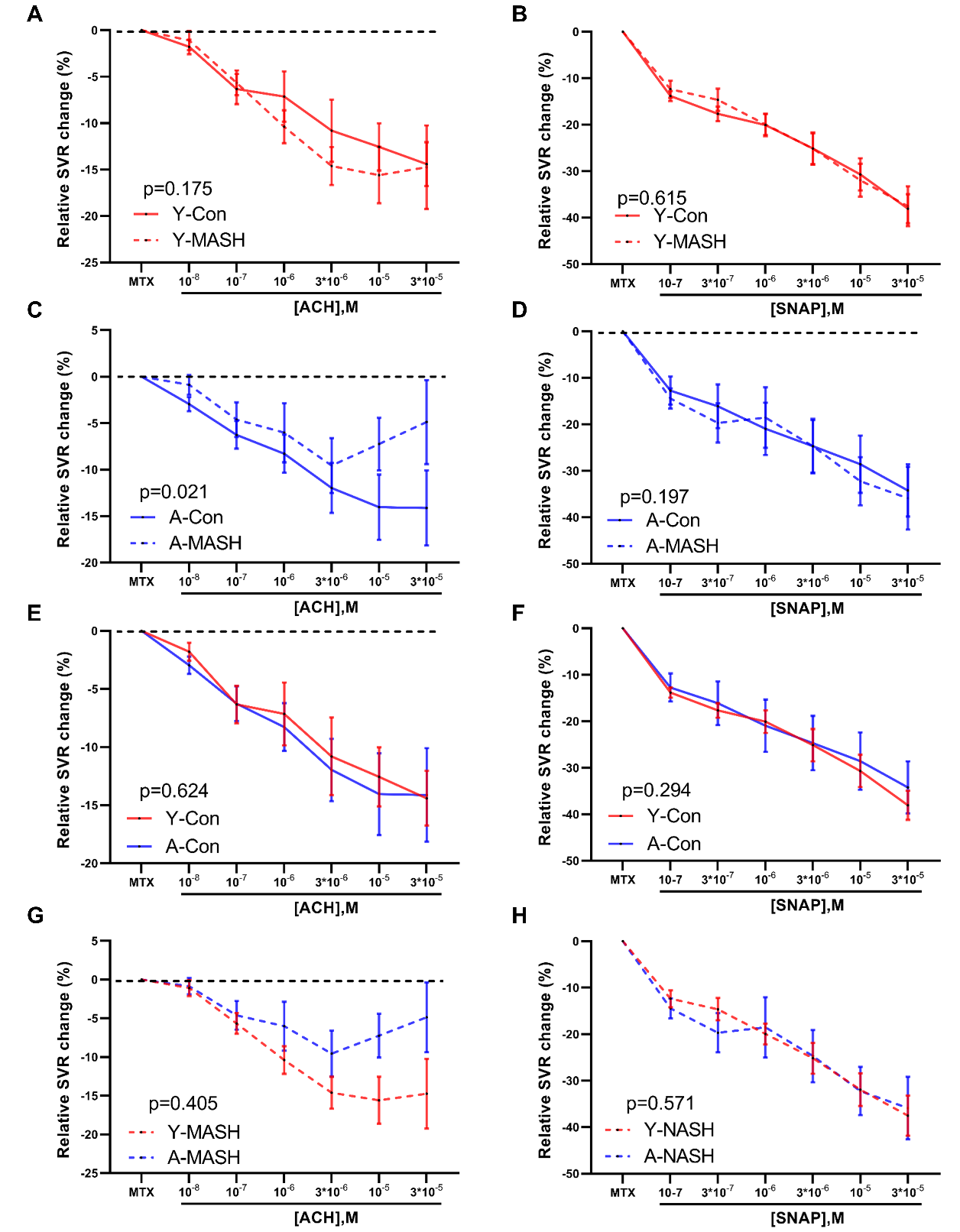


**Supplementary figure 9.** **MASH-associated changes in the sinusoidal vascular bed reactivity with biological aging.** The relative SVR change in response to increasing concentrations of (A, C, E, and G) ACH and (B, D, F, and H) SNAP administration in the portal vein of young (8 weeks) and aged (78 weeks) rats after 12 weeks of MCD diet or standard chow diet feeding. Data expressed as mean ± SEM (N = 9-12), general linear model for repeated measurements. SVR, sinusoidal vascular resistance; MTX, methoxamine; ACH, acetylcholine; SNAP, S-Nitroso-N-acetyl-DL-penicillamine.


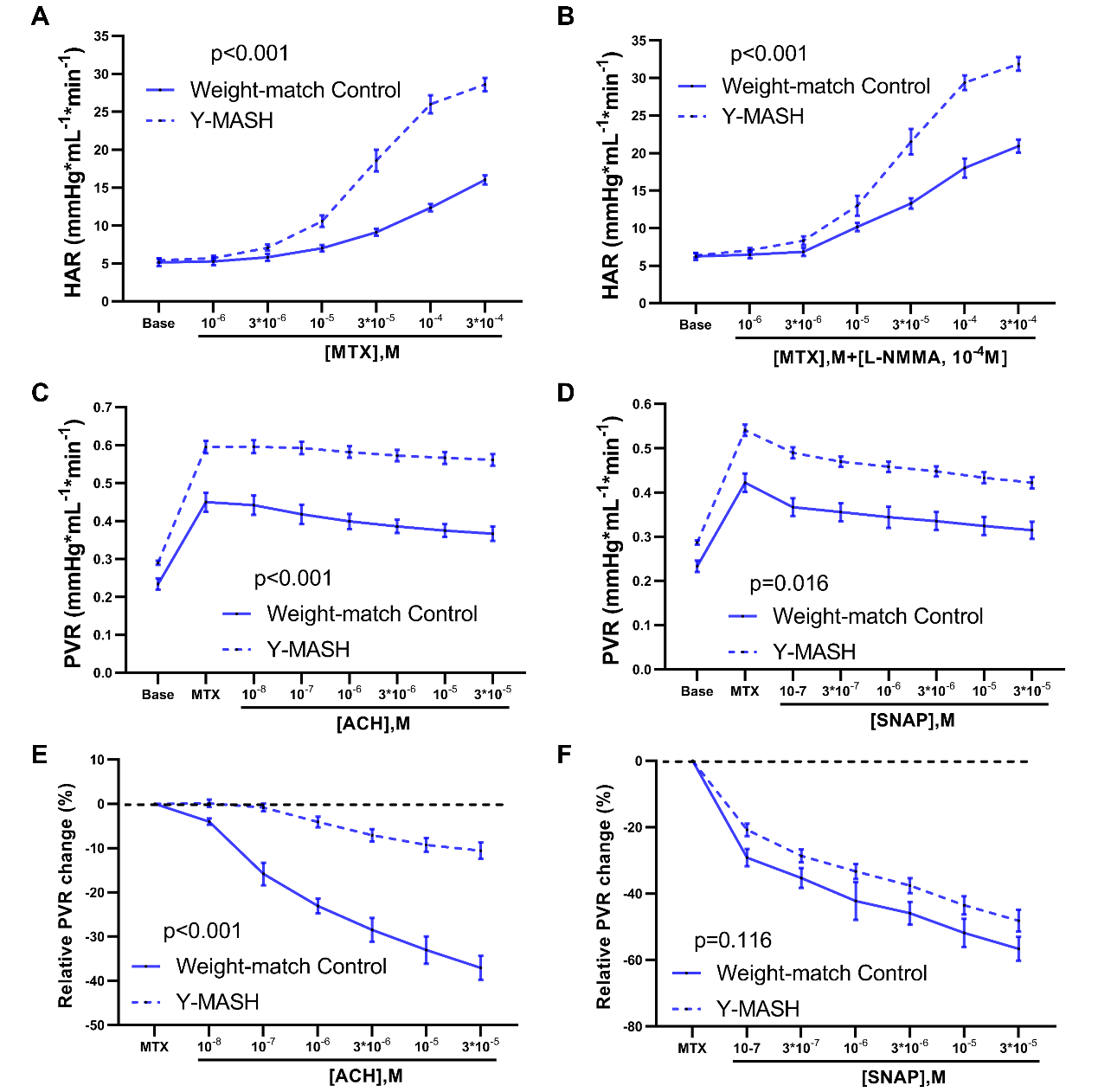


**Supplementary figure 10. Vascular bed reactivity of young MASH rats in comparison to weight-matched control rats.** Absolute values of HAR of young MASH rats and weight-match control rats in response to increasing concentrations of MTX in the (A) absence (B) and presence of L-NMMA. The absolute values of PVR and their relative changes in response to increasing concentrations of (C and E) ACH and (D and F) SNAP of young MASH and weight-match control rats. Data expressed as mean ± SEM (N = 9-12), general linear model for repeated measurements. HAR, hepatic arterial resistance; MTX, methoxamine; L-NMMA, NG-Methyl-L-arginine acetate salt; PVR, portal venous resistance; ACH, acetylcholine; SNAP, S-Nitroso-N-acetyl-DL-penicillamine.


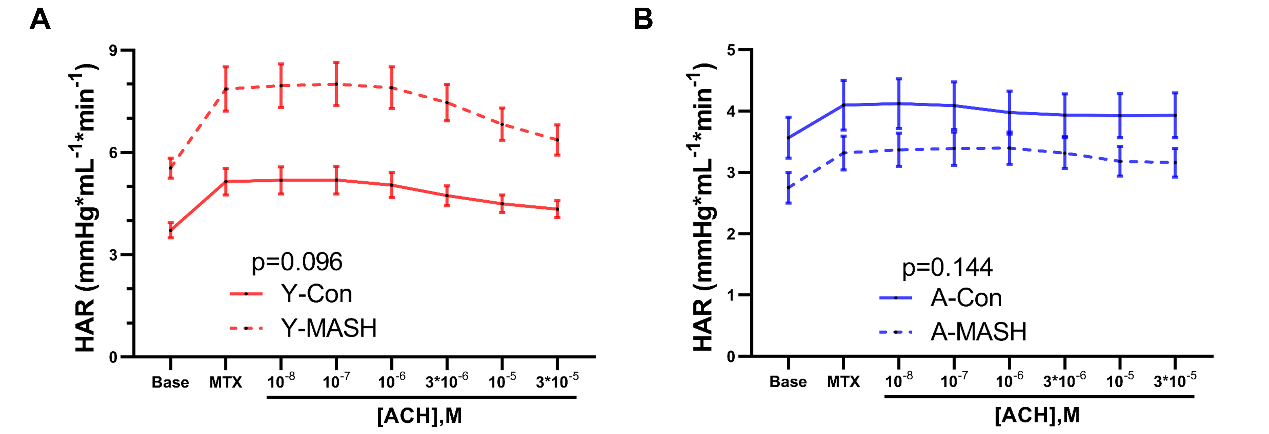


**Supplementary figure 11. Age- and MASH-associated changes in HAR in response to ACH.** The absolute value of HAR in response to increasing concentrations of ACH administration in the portal vein of (A) young cohort and (B) aged cohort of rats fed with standard chow diet or MCD diet. Data expressed as mean ± SEM (N = 9-12), general linear model for repeated measurements. HAR, hepatic arterial resistance; MTX, methoxamine; ACH, acetylcholine.
